# Synergistic Potential of Organotin(IV) Carbodithioate Derivatives with Vitamins D and E in MCF-7 and MDA-MB-231 Breast Cancer Cells

**DOI:** 10.3390/ph19040571

**Published:** 2026-04-02

**Authors:** Balquees Kanwal, Farzana Shaheen, Syeda Saba Shah, Yasmeen Cheema, Saqib Ali, Rumeza Hanif

**Affiliations:** 1 Atta-ur-Rahman School of Applied Biosciences (ASAB), National University of Sciences and Technology (NUST), H-12, Islamabad 44000, Pakistan; 2 Department of Chemistry, Faculty of Science, Allama Iqbal Open University, H-8 Campus, Islamabad 44310, Pakistan; 3 Department of Chemistry, Quaid-i-Azam University, Islamabad 45320, Pakistan

**Keywords:** organometallic compounds, nutraceuticals, in silico modeling, protein–ligand interactions, anticancer potential, novel therapeutic approach

## Abstract

**Background**: Breast cancer (BC) remains the most prevalent malignancy among women worldwide, with one in eight at risk during their lifetime. Platinum-based chemotherapeutic drugs, despite of their binding to the DNA of cancer cells, are plagued by toxicity and resistance, necessitating the need for safer and more effective alternatives, such as organometallic complexes. Both synthetic organometallic complexes and natural compounds have attracted attention in this regard. Organotin(IV) complexes are promising chemotherapeutics due to their structural versatility and bioactivity, while vitamins such as Vitamin D (VD) and Vitamin E (VE) exhibit antiproliferative, anti-inflammatory, and antioxidant properties, making them valuable candidates for combination therapy. **Methodology**: In this study, six novel organotin(IV) dithiocarbamate complexes [LMe_3_Sn (Complex 1), LBu_3_Sn (Complex 2), LPh_3_Sn (Complex 3), LMe_2_SnCl (Complex 4), LBu_2_SnCl (Complex 5), and L_2_Me_2_Sn (Complex 6), where L = (E)-4-styrylpiperazine-1-carbodithioate], were synthesized and characterized by FT-IR, ^1^H-, ^13^C-NMR, and elemental analysis. **Results**: Structural studies confirmed penta- and hexacoordination geometries. In silico docking against six BC-related proteins identified Complexes 2 and 4 with both vitamins as promising candidates, exhibiting strong binding affinities, with stable interaction profiles. However, integration of pharmacokinetic, antioxidant, and anti-inflammatory analyses highlighted Complex 4 with both vitamins as the most potent candidate owing to its superior ADME characteristics and balanced biological properties. Subsequent in vitro assays confirmed these findings, as Complex 4 demonstrated strong cytotoxic activity against both MCF-7 (>1.16-fold) and MDA-MB-231 (>1.46-fold) cell lines, surpassing the efficacy of cisplatin. Remarkably, co-administration of VD or VE with Complex 4 further enhanced its anticancer potential, with Chou–Talalay combination index values < 1 (0.66–0.91) indicating a synergistic interaction. **Conclusions**: Collectively, these results identify Complex 4 as a promising lead compound, and its synergistic activity with natural vitamins may promote cell death, likely through apoptosis induction and modulation of oxidative stress, underscoring its potential as an effective and less toxic therapeutic strategy for breast cancer management.

## 1. Introduction

Breast cancer (BC) remains the foremost diagnosed malignancy affecting women globally, representing approximately 23% of all cancer incidences worldwide. The World Health Organization’s 2022 report underscores the staggering impact of this disease, citing an annual incidence of 2.31 million new cases reported globally [1]. BC ranks highest in both incidence and mortality rates among women worldwide [2]. This highly heterogeneous disease comprises subtypes with distinct biological behaviors and prognostic profiles [3]. The prevalent shortcomings of commercially available chemotherapeutic agents against BC pose substantial challenges such as poor bioavailability, high doses resulting in adverse systemic effects, non-specific target, and the emergence of multi drug resistance [4,5]. While targeted therapies like monoclonal antibodies and endocrine treatments show promise by mitigating systemic harm, they encounter setbacks due to the rapid onset of therapeutic resistance observed in a majority of patients shortly after treatment initiation [6,7]. These limitations highlight the necessity of alternative approaches which not only reduce the tumor mass but can enhance the therapeutic targeting and decrease side effects. Combination strategies which combine conventional or experimental agents with supportive nutraceuticals such as vitamins emerged as an attractive area to deal with these challenges.

Among nutraceuticals, Vitamins D (VD) and E (VE) have received notable attention for their antitumor potential. VD is involved in cell differentiation, proliferation inhibition, and potential for reducing BC risk [8]. Similarly, VE and its isoforms are mainly associated with tumor metabolism, proliferation, metastasis, invasion, and chemoresistance [9,10]. Preclinical and clinical studies reported that sufficient serum levels of both vitamins are linked to reduced risk of BC and better treatment responses [11,12]. Although their benefits are limited as monotherapies, accumulating studies suggest that they have better effects when used in combination with other chemotherapeutic drugs. This makes them an attractive option for synergistic studies with other anticancer drugs.

Organotin(IV) complexes have emerged as potential biologically active therapeutic agents [13] because of their ability to adopt various structural patterns and geometries derived from vacant d-orbitals [14]. Moreover, their capability to target biomolecules like DNA and proteins disrupts cancer cell proliferation and triggers apoptosis [15]. These non-platinum chemotherapeutic complexes have the ability to exhibit fewer side effects, increased antiproliferative actions, higher excretion abilities, and reduced toxicities compared to other platinum-based chemotherapeutic drugs [13]. Although previous studies have demonstrated the roles of organotin(IV) complexes in different cancer models, their thorough application in BC remains unexplored. Notably, their mode of action overlaps with both VD and VE’s mechanism of action in modulating immune response, apoptosis, inflammation, and oxidative stress, indicating that combining them can result in synergistic therapeutic benefits.

**Figure 1:**
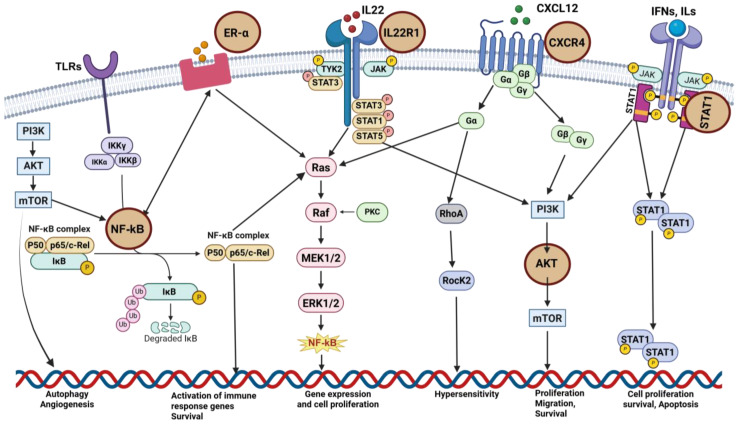
The figure illustrates the key signaling molecules involved in the progression of breast cancer, including ERα, CXCR4, IL22R1, Akt1, NF-kβ, and STAT1 (shown in brown circles). These proteins have significant contributions in various stages of tumorigenesis, like cell survival, proliferation, and metastasis. Alterations or dysregulations of these signaling pathways lead to tumor aggressiveness and resistance to therapy.

BC progression and therapeutic resistance are intricately regulated by diverse signaling networks that influence tumor initiation, growth, survival, and metastasis. Dysregulation of these signaling molecules contributes to tumor aggressiveness and plays a significant role in the development of treatment resistance [16]. Six proteins, namely, CXCR4, ERα, IL-22R1, AKT1, NF-κB, and STAT1, are particularly relevant as they are involved in tumor initiation, growth, metastasis, immune evasion, and resistance to therapy [17,18,19,20,21,22]. A schematic representation of their contribution in BC progression is shown in Figure 1. Evaluating the interactions of organotin(IV) complexes and vitamins with these proteins gives mechanistic insights into the effect of these compounds on them. Consequently, molecular docking studies were performed using these selected proteins to evaluate the binding affinities of organotin(IV) complexes and vitamins to rationalize their corresponding biological effects.

Although previous studies have demonstrated the individual roles of organotin(IV) complexes, VD, and VE in cancer treatment, their combined impact on BC has not been thoroughly explored. The interactions of these compounds with the proteins responsible for building resistance at the molecular level is not well understood previously. In this study, six novel (E)-4-styrylpiperazine-1-carbodithioate organotin(IV) complexes were synthesized, characterized, and investigated for their antiproliferative properties alone or in combination with VD and VE on BC cell lines. In silico docking and pharmacokinetic profiling were carried out against BC-related proteins, followed by in vitro validation on two BC cell lines, MCF-7 and MDA-MB-231, to assess the efficacy of these combinations. Our findings demonstrate the therapeutic potential of combining organotin(IV) complexes with vitamins as a promising strategy for improving the outcomes of BC treatment.

## 2. Results and Discussion

### 2.1. Synthesis of Organotin(IV) Complexes

**Scheme 1:**
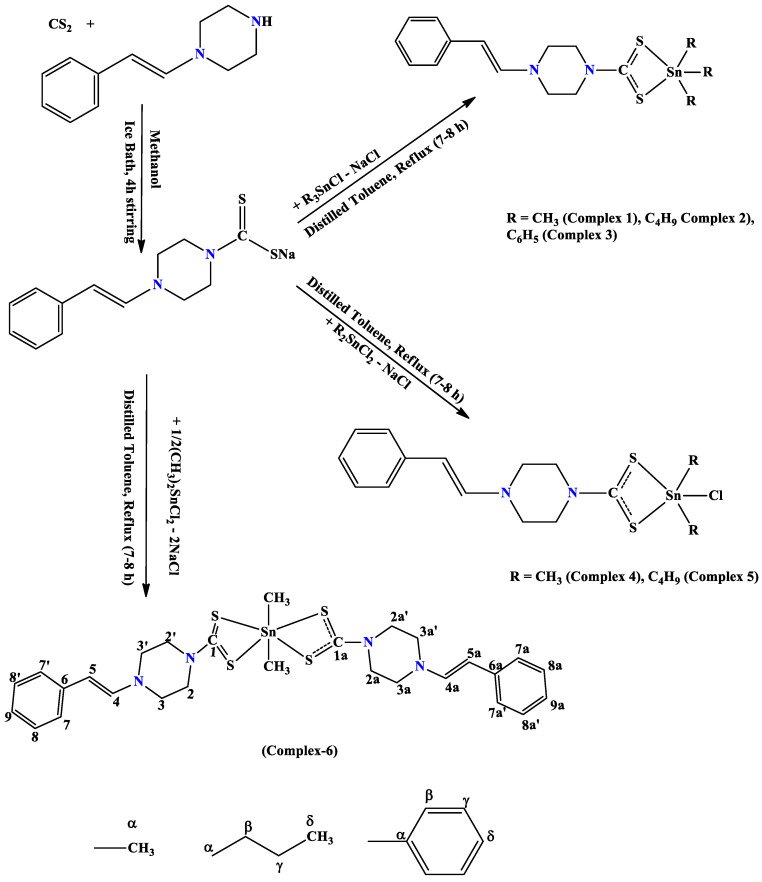
Synthesis of the ligand and complexes (1–6) along with the atom numbering pattern for NMR spectral interpretation. (The protons on the left-hand side are chemically equivalent to those on the right-hand side, e.g., 1 = 1a, 2 = 2a, and so on).

Organotin(IV) carbodithioate derivatives (1–6) and ligand [Sodium(E)-4-styrylpiperazine-1-carbodithioate] were synthesized and characterized. Scheme 1 offers a schematic representation of the synthesis of sodium salt of ligand and organotin(IV) complexes.

### 2.2. FT-IR Spectral Data

All derivatives (1–6) of Sodium(E)-4-styrylpiperazine-1-carbodithioate exhibited characteristic bands in the infrared region (Figures S1–S7). The bands observed at 533–549 cm^−1^ and 351–360 cm^−1^ were assigned to Sn–C and Sn–S stretching vibrations, respectively. These values closely matched those reported for various organotin(IV)–sulfur donor ligands, providing supporting evidence. In addition, a distinctive band at 315 cm^−1^ and 319 cm^−1^ was observed in chlorodiorganotin(IV) derivatives 4 and 5, which can be attributed to Sn-Cl vibrations [23]. Symmetric and asymmetric CSS stretching vibrations in FT-IR spectroscopy are useful parameters to understand their coordination chemistry. The difference between asymmetric ν_asym_(CSS) and symmetric ν_sym_(CSS) stretching vibrations gave information about the coordination behavior of dithiocarbamate ligands. The symmetric CSS vibrations were observed in the range of 983–975 cm^−1^, while the asymmetric stretching vibrations were present in the range of 1118–1140 cm^−1^. According to reports in the literature, a difference of less than 200 cm^−1^ serves as an indication of a bidentate coordination mode, whereas a difference exceeding 200 cm^−1^ suggests monodentate coordination [24]. The calculated Δν value (Δν = ν_asym_CSS − ν_sym_CSS) was in the range of 131–147 cm^−1^, indicating that dithiocarbamates coordinate with the tin atom in a bidentate mode. This finding is consistent with previously reported similar complexes [25]. Replacement of symmetric and asymmetric stretch with a sharp singlet at around 1000 cm^−1^ in complexes signifies the symmetrical coordination of the dithiocarbamate moiety to the metal ions. Conversely, the splitting of the identical band within a range of 20 cm^−1^ in the same region is attributed to the monodentate binding mode of the dithiocarbamate ligand [26]. In this study, no such splitting was observed, which shows that the ligand is bound to the tin center asymmetrically. Hence, Complexes 1–5 exhibit penta-, while Complex 6 exhibit hexa-coordination.

Regarding the C-N stretching vibrations, all complexes exhibited bands in the range of 1473–1487 cm^−1^. This range fell between the reported values for C-N single bonds (1250–1360 cm^−1^) and C=N double bonds (1640–1690 cm^−1^). This suggested a partial double bond character in the C-N bonds within these complexes [27]. Overall, the observed spectral characteristics provided valuable insights into the molecular structure and bonding properties of the derivatives of sodium(E)-4-styrylpiperazine-1-carbodithioate.

### 2.3. UV–Visible Spectroscopy

The electronic spectra of organotin(IV) dithiocarbamate complexes reveal three fundamental bands associated with the (C=N) bond, the electron pair of sulfur, and the metal-ligand (M-L) bond in the UV–Visible absorption spectra. In the UV–Visible spectrum of the ligand, a prominent band was observed at 259 nm, attributed to the π–π* transition of the N=C=S group. Upon complexation, this absorption band undergoes a shift to lower wavelengths, falling within the range of 251–257 nm (Figure S8). This spectral shift aligned with the reported literature which indicated that the complexation-induced involvement of the chromophore group NCS_2_ leads to a shift in the absorption band towards lower wavelengths [26]. A shoulder band of weak intensity in the ligand and complexes due to n–π* transition was observed around 180 nm. An additional shoulder band in the case of all complexes was observed due to the M-L charge transfer transition around 300 nm. This absorption feature suggested the presence of an extended conjugation system, as it arises from the electronic transition between the p-orbital of sulfur and the 5d-orbital of the tin metal [28].

All six complexes displayed highly similar patterns in the UV–Visible spectrum, with only minor variations in the π–π* transition region. The main ligand band shifted from 259 nm to 251–257 nm upon complexation in every case, showing only slight differences among the complexes. The n–π* transition around 180 nm and the M–L charge-transfer shoulder near 300 nm appeared at nearly identical positions for all six complexes. Thus, except for small shifts within the π–π* band, the UV–Visible spectra of the six complexes are essentially similar, indicating comparable electronic environments and coordination behavior.

### 2.4. NMR Spectroscopy

The ligand salt and the complexes were analyzed using proton NMR spectra recorded as solutions in deuterated chloroform (CDCl_3_). The signals for all protons in the complexes were identified, and the presence of alkyl and phenyl protons in their respective regions confirmed complex formation. The number of protons was calculated using the integration method, and the results are in good agreement with the proposed structure. The R groups exhibited signals in the expected region (Figures S9–S22) [25].

Sharp singlets were observed for methyl protons attached to the tin atom in 1, 4, and 6 at 0.69, 1.29, and 0.84 ppm, respectively, with well-defined satellite peaks due to ^119^Sn–^1^H coupling. From observed coupling constant ^2^*J* [^119^Sn–^1^H] values, the C–Sn–C angle (θ) was found using Lockhart’s equation (θ = 0.0106[^2^*J*]^2^ − 1.32[^2^*J*] + 133.4) [29,30]. Both ^2^*J* and θ values gave useful information about the geometry around the tin atom in the solution. The ^2^*J*[^119^Sn–^1^H] values obtained for Complexes 1 and 6 were 56 Hz and 60 Hz, respectively. These values were normally in the range expected for four-coordinate tin [31] and consistent with C–Sn–C angles (θ) of 110.1° and 111.8°, which suggests that they have a tetrahedral geometry. The ^2^*J*[^119^Sn–^1^H] value obtained for 4 was 81, which corresponds to an angle value of 131.4° and therefore a distorted trigonal bipyramid geometry. Protons of n-butyl in 2 and 5, and phenyl attached to tin in 3 gave a complex signal pattern and were assigned according to the previous literature [31]. Due to such a complicated signal pattern, no ^119^Sn–^1^H coupling was observed.

Similarly, ^13^C-NMR spectral analysis for all the signals obtained was assigned by comparing it with the previous literature. The ligand and all complexes gave signals in the expected regions. Complex 1 formation was further confirmed by the ^119^Sn–^13^C coupling constant value obtained as 354 ppm. From the ^1^*J*[^119^Sn,^13^C] values, C–Sn–C angles (θ) were found using Lockhart’s equation (^1^*J*[^119^Sn,^13^C] = 11.4θ − 875). Complex 1 exhibits a tetrahedral geometry, with a coupling constant value being consistent with an angle value of 108.0°. This shows that the ligand is dissociated in the solution state and coordinated via only one sulfur atom. Complex 2 has 349.2 Hz, 20.9 Hz, and 68.0 Hz coupling constant values for ^1^*J*(^119^Sn–^13^C), ^2^*J*(^119^Sn–^13^C), and ^3^*J*(^119^Sn–^13^C) coupling satellites, respectively, indicating that the complex has a four-coordinate quasi-tetrahedral geometry around tin centers [32] in the solution state (Figure S13).

To further confirm the coordination environment around the tin center, representative complexes were subjected to ^119^Sn NMR spectra. The chemical shift values obtained were 29.7 and 58.5 for Complexes 2 and 3, respectively, indicating a tetrahedral geometry. On the other hand, a chemical shift value of −144.56 ppm for Complex 5 corresponded to a trigonal bipyramid geometry, as reported in the previous literature [33]. ^13^C-NMR and ^1^H-NMR spectra of Complex 2 are shown in Figures S13 and S14, respectively.

### 2.5. Differential Expression Pattern of Proteins

To evaluate the differential expression of selected BC-associated proteins, the GEPIA tool was used for comparing protein expression levels in both tumor and normal tissue samples. The fold change mentioned below indicates the relative overexpression (>1) or downregulation (<1) of each protein in tumor samples compared to control. Box-plot analysis through the GEPIA tool revealed that all proteins showed differential expression except NF-ĸB. Specifically, CXCR4 (~2.4 fold change), AKT1 (~1.5 fold change), and STAT1 (~2.8 fold change) were overexpressed, whereas ER-α (~0.3 fold change) and IL-22R (~0.7 fold change) were significantly downregulated. Interestingly, NF-ĸB showed no significant difference in expression between the two groups, suggesting its possible involvement in regulating other proteins or signaling pathways rather than directly contributing to the development of BC. The overexpression of CXCR4 is consistent with a meta-analysis study which reported that increased expression of CXCR4 correlates with poor overall survival and disease-free survival in BC patients [34]. However, the upregulation of STAT1 and downregulation of ER-α are in contrast with the study of Hou et al., which reported that STAT1 is important in transcriptional regulation of ER-α, and depletion of STAT1 decreases the levels of the ER-α protein [35].

Furthermore, the prognostic value by overall survival analysis showed that a low expression of AKT1 and ER-α and a high expression of CXCR4, IL-22R, and STAT1 significantly increased a patient’s survival probability in BC (Figure S23). As from the results of the GEPIA analysis, the log-rank *p* values did not reach statistical significance (*p*-value < 0.05), it is noteworthy that the expression levels of the proteins examined in this study were significantly altered in relation to the survival outcome. These observed alterations in protein expression provided valuable insights into the potential involvement of these proteins in the underlying mechanisms of BC. Our differentially expressed proteins could also potentially serve as biomarkers for the survival outcome being studied. Our results suggested that these proteins warrant further investigation to understand their functional roles and potential implications in the context of the survival outcome.

### 2.6. Construction of Protein–Protein Interaction Map

A PPI map was constructed for all six proteins (Figure S24). Our network showed 11 nodes, 42 edges, a 7.64 average node degree, an average local clustering coefficient of 0.899, and an enrichment *p*-value of 1.84 × 10^−7^, signifying strong interactions. According to the results of our GEPIA analysis, NF-ĸB expression did not change in BC compared to the control. STRING analysis found that NF-ĸB has strong interactions with AKT1 and ER-α with an overall score of 0.958 and 0.839, respectively. This result may indicate that NF-ĸB is involved in breast cancer signaling pathways, as reported by Li et al., that AKT1 is involved in NF-ĸB activation by phosphorylating IκB Kinase (IKK) [36].

### 2.7. Molecular Docking Analysis

Molecular docking of the organotin(IV) complexes, VD, and VE against six selected BC–related proteins was performed to evaluate their binding affinities and interaction patterns. The corresponding binding energies, interacting residues, and nature of molecular interactions are summarized in Table S1. The most favorable docking poses were identified based on the lowest binding energies, which were considered indicative of the highest binding affinity. Representative complexes with the strongest interactions are summarized in Table S2 (highlighted in red color). Beyond binding energies, interaction profiling revealed that key stabilizing forces included hydrogen bonds, hydrophobic contacts, π–sulfur interactions, and π–π stacking, all of which contributed to the specificity and stability of ligand–protein complexes [37]. These interactions played a significant role in enhancing the stability and specificity of ligand–protein binding.

Interaction analysis was performed using PLIP and BDS, which identified all possible ligand–protein interactions. Additionally, 2D interaction diagrams were generated through the LigPlot server (PDBsum) to further visualize binding profiles (www.ebi.ac.uk/thornton-srv/databases/pdbsum/Generate.html, accessed on 23 October 2025).

In the current study, molecular docking was conducted to investigate the interaction of various complexes with the activation loop of different proteins.

Complex 1 demonstrated strong bonding and non-bonding interactions with amino acid residues of the activation loop of STAT1, i.e., Gln-1272, Gln-352, Asn-1355, Met-392, Glu-394, Lys-297, Glu-1157, Lys-1161, His-1158, Met-1154, Lys-1150, Tyr-1356, His-1158, and Tyr-256. Complex 1 showed hydrogen bonding with Met-392, hydrophobic interactions with Lys-297, Met-392, Glu-1157, and Lys-1161, and Pi–alkyl interactions with Lys-1161 and His-1158 (Figure S25). Complex 2 displayed strong bonding and non-bonding interactions with amino acid residues of the activation loop of AKT1, i.e., Leu-295, Asp-274, Gly-311, Glu-198, Asp-292, His-194, Phe-161, and Glu-191. Complex 2 showed hydrogen bonding with Glu-394, hydrophobic interactions with Phe-161, His-194, Leu-295, and Glu-393, and attractive charge interactions with Glu-393 (Figure S26). Complex 3 showed strong bonding and non-bonding interactions with amino acid residues of the activation loop of IL22R, i.e., Asn-176, Ile-159, His-161, Thr-207, Lys-44, Asp-162, and Lys-182. Complex 3 had hydrogen bonding with Lys-44, hydrophobic interactions with Lys-44, His-161, and Asp-162, and Pi–cation and Pi–alkyl interactions with Lys-44 (Figure S27). Complex 4 exhibited strong bonding and non-bonding interactions with the amino acid residue of the activation loop of CXCR4, i.e., Trp-94, Asp-187, Arg-188, His-281, Ile-284, Glu-288, and Ser-285. Complex 4 showed hydrogen bonding with His-113, hydrophobic interactions with Ile-259 and Ile-284, Pi–sulfur interaction with His-113, Pi–alkyl interaction with Trp-94, and attractive charge interaction with Asp-187 (Figure S28). Complex 5 had strong bonding and non-bonding interactions with the activation loop of ER-α, i.e., Met-343, Met-421, His-524, Met-528, trp-383, and Thr-347. Complex 5 demonstrated hydrophobic interactions with Leu-387, Phe-404, Ile-424, and Leu-525, Pi–sulfur interactions with Trp-383, and Pi-alkyl bonding with Lleu-391 and Met-421 (Figure S29). Complex 6 showed strong bonding and non-bonding interactions with amino acid residues of the activation loop of CXCR4, i.e., His-281, Cys-28, Asp-262, Val-196, Gln-200, Arg-188, Asp-186, Ile-187, Arg-30, Glu-288, Ser-285, and Trp94. Complex 6 had hydrogen bonding with Arg-188, hydrophobic interactions with Arg-30, Glu-31, Val-196, His-281, and Lys-282, sulfur interaction with Asp-187, and attractive charge interaction with Asp-187 (Figure S30). VD displayed strong bonding and non-bonding interactions with amino acid residues of the activation loop of STAT1, i.e., Asn-1355, Asn-1357, Gln-1352, Glu-353, Lys-278, Gln-1272, Glu-268, Val-153, Ser-269, Lys-150, Gln-271, Gln-275, Tyr-1356, and Arg-274. VD showed hydrogen bonding with Asn-1357, hydrophobic interactions with Lys-150, Val-153, Glu-268, Gln-271, Gln-272, and Tyr-1356, and alkyl interactions with Arg-274 (Figure S31). VE demonstrated strong bonding and non-bonding interactions with amino acid residues of the activation loop of CXCR4, i.e., Gly-159, Trp-125, Ile-213, Leu-210, Val-206, Leu210, Trp-125, Pro-163, Leu-167, and Ile-213. VE showed hydrogen bonding with Gly-159, hydrophobic interactions with Trp-125, Leu-167, Val-206, Leu-210, and Leu-213, Pi–pi interaction with Trp-125, and pi–alkyl interaction with Ile-213 (Figure S32).

### 2.8. Molecular Docking Simulations

To evaluate the stability of the protein–ligand complex, MD simulations were performed on the CABS-flex 2.0 server. The alterations induced in the protein through the ligand can be assessed by MD simulations. The root mean square fluctuation (RMSF) profiles of the selected proteins (CXCR4, ERα, IL-22R1, AKT1, NF-κB, and STAT1) produced by CABS-flex represent the amino acid flexibility. A higher value of RMSF predicts more flexibility, whereas lower values indicate the restricted motion of the system through the simulation course [38]. The CABS-flex server generates an output file consisting of 10 modeled structures on submission of the protein structure in PDB format with default parameters. It generates a graph of the RMSF profile for the calculation of per-residue fluctuation in the protein complex.

Results from CABs-flex demonstrated that Complexes 2 and 4 with vitamins showed the best fluctuation poses with proteins AKT and CXCR4, respectively.

**Figure 2:**
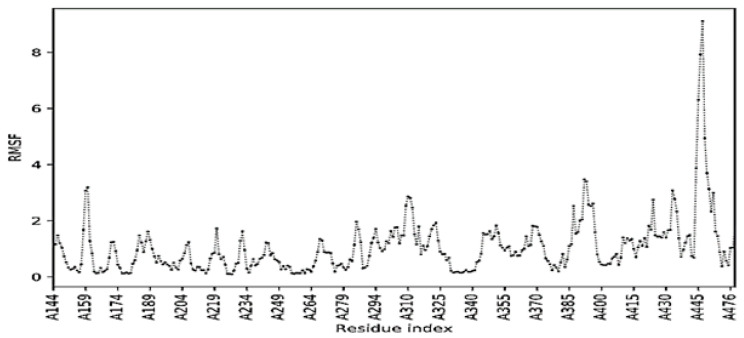
Root mean square fluctuation (RMSF) plot of the AKT protein complex.

**Figure 3:**
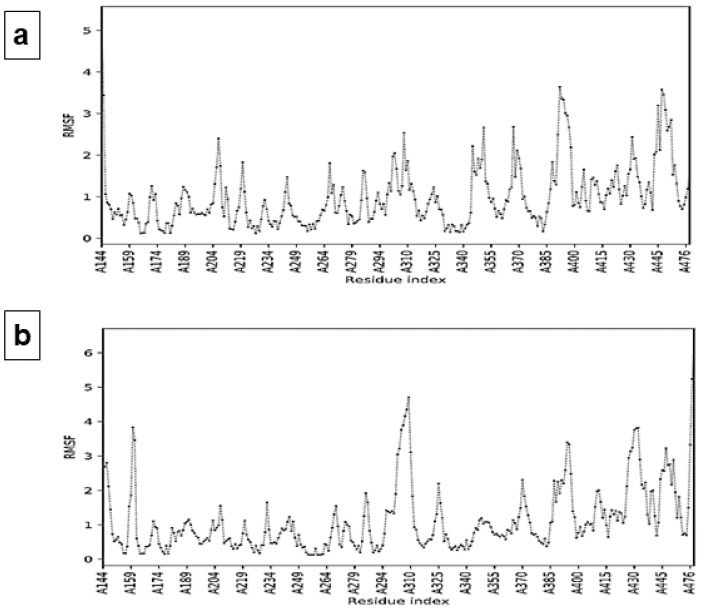
Root mean square fluctuation plots of AKT protein with (**a**) Complex 2 and (**b**) VD.

**Figure 4:**
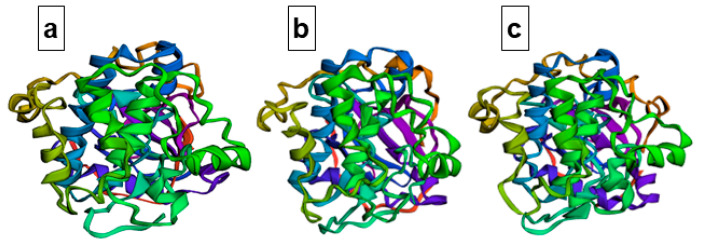
Stable structure generated after MD simulations: (**a**) AKT protein, (**b**) AKT with Complex 2, and (**c**) AKT with VD.

The best stable configurations of the proteins were generated by MD simulations from the CABS-flex server [37]. After performing simulations, it was found that the proteins had helices and many loops. Stable structures of the AKT protein alone or docked with Complex 2 and VD are shown in Figure 2 and Figure 3. Stable structures were generated after MD simulations, as shown in Figure 4.

The AKT protein showed maximum fluctuation at A.A residue 447 as 9.10 Å, and minimum fluctuation at A.A residue 227 measuring 0.09 Å (Figure 2). On the other hand, Complex 2 had maximum fluctuation at A.A residue 392 with a value of 3.64 Å and minimum fluctuation at A.A residue 340 measuring 0.159 Å with the AKT protein. Similarly, VD exhibited maximum fluctuation at A.A residue 309 as 4.70 Å and minimum fluctuation at A.A residue 260 as 0.125 Å, signifying a lower RMSF value and more stability (Figure 3).

**Figure 5:**
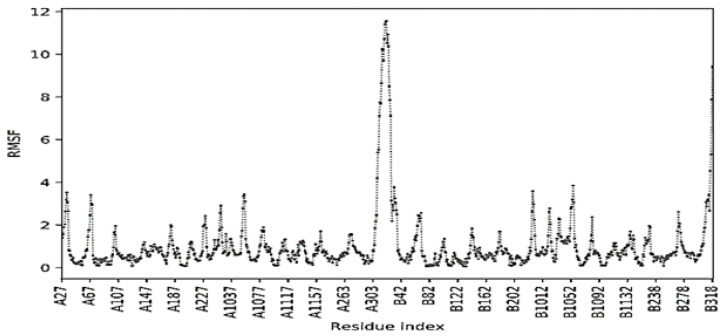
Root mean square fluctuation (RMSF) plot of the CXCR4 protein complex.

**Figure 6:**
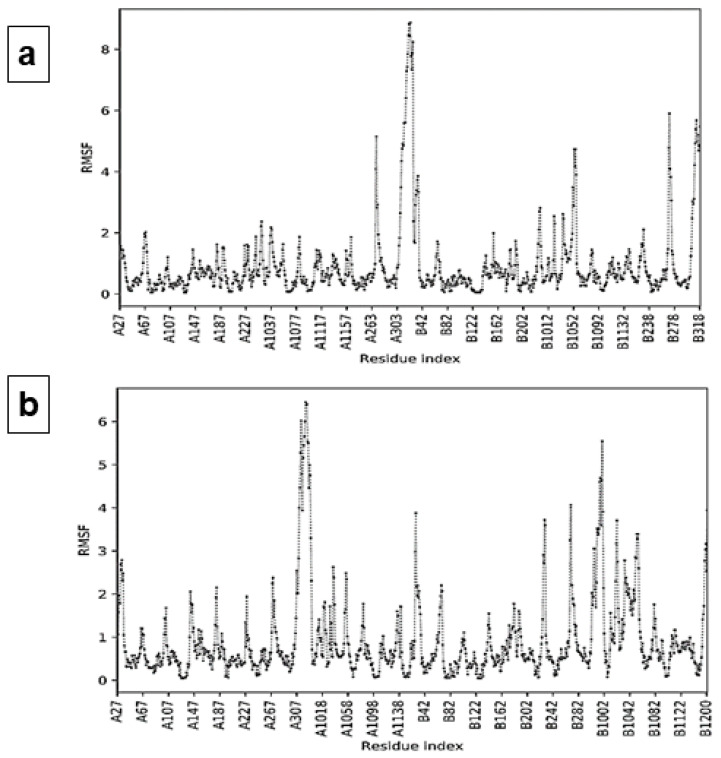
Root mean square fluctuation plots of CXCR4 protein with (**a**) Complex 4 and (**b**) VE.

**Figure 7:**
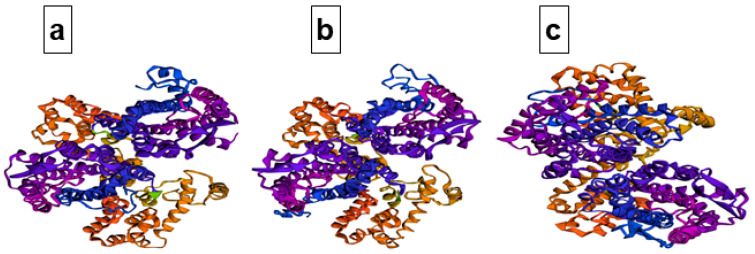
Stable structure generated after MD simulations: (**a**) CXCR4 protein, (**b**) CXCR4 with Complex 4, and (**c**) CXCR4 with VE.

CXCR4 showed maximum fluctuation at A.A residue 320 measuring 11.40 Å and minimum fluctuation at A.A residue 200 with a value of 0.09 Å (Figure 5). Complex 4 showed maximum fluctuation with CXCR4 at A.A 322 as 8.83 Å and minimum fluctuation at A.A residue 1063 as 0.069 Å. Similarly, VE displayed maximum fluctuation at A.A residue 321 as 6.45 Å and minimum fluctuation at A.A residue 130 measuring 0.04 Å, signifying lower RMSF values of docked complexes compared to the single protein and more stable complexes (Figure 6). Stable structures of the CXCR4 protein alone or docked with Complex 4 and VE are shown in Figure 7.

### 2.9. Drug Likeliness of Compounds Through Swiss ADME Server

ADME profiling determines whether potential inhibitors are biologically active, as inhibitors with poor ADME properties and high toxicity effects on biological systems are the primary causes of most medicines’ failure in preclinical and clinical phases [39].

ADME profiles and drug likeness analysis revealed that organotin(IV) Complexes 1–5 have shown high gastrointestinal absorption, whereas Complex 6 and both vitamins displayed low predicted absorption. Moreover, Complexes 1, 3, and 4 were predicted to allow the crossing of the blood–brain barrier, which could be useful in targeting brain metastasis in BC, although it can raise concerns regarding central nervous system toxicity [40]. Efflux prediction via P-glycoprotein (P-gp) substrate analysis showed that only Complexes 1, 4, and VD did not appear in the analysis and may achieve higher cellular retention.

Regarding drug likeness, Complexes 1 and 4 suggested most favorable drug properties with no violations of Lipinski’s rule of five. Regarding the bioavailability score (BA), except for Complexes 2 and 3, all the complexes had a moderate score, predicting good oral bioavailability. Collectively, these findings suggest Complexes 1 and 4 as favorable candidates in terms of drug likeness and balanced pharmacokinetic profiles. Results of ADME properties of our organotin(IV) series of complexes and vitamins are shown in Table S3.

Bioactivity prediction/physiochemical through Molinspiration server:

The toxicity prediction, bioactivity, and physiochemical properties of complexes evaluated by Molinspiration software (www.molinspiration.com) are shown in Table S4. The analysis confirmed that both Complexes 1 and 4 satisfied Lipinski’s rule, supporting their favorable bioavailability and absorption profiles, in line with ADME predictions and the previous study [41]. Complexes 5, 6, and vitamins also displayed favorable results with the only violation. Notably, bioactivity scores indicated that Complexes 1 and 4 had favorable predicted activity, further supporting their potential as drug-like candidates.

### 2.10. Toxicity Prediction Through Protox-II Server

Toxicity evaluation using the Protox-II server predicted oral LD50 values ranging from 264 to 5000 mg/kg for the tested complexes, as shown in Table S5. Among all, VE showed the highest LD50, indicating the lowest predicted acute toxicity (prediction accuracy: 70.9%), while Complex 2 exhibited the lowest LD50, suggesting comparatively higher toxicity. Complexes 1, 3, 4, and 6 were classified as toxicity class 4 and were predicted to be inactive for hepatotoxicity, carcinogenicity, immunotoxicity, mutagenicity, and cytotoxicity. However, the relatively low prediction accuracy (23%) highlights the need for experimental validation of these in silico toxicity results.

### 2.11. Results of Biological In Vitro Assays

#### 2.11.1. Antioxidant Activity

**Figure 8:**
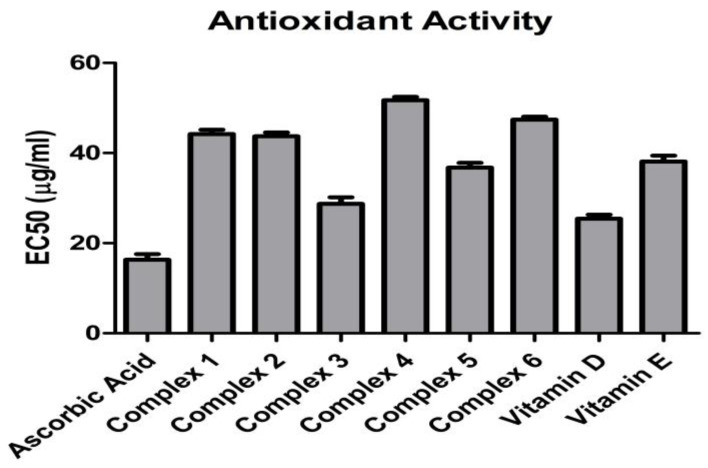
EC50 values representing the antioxidant activity of organotin(IV) complexes and vitamins, determined using the DPPH free radical scavenging assay. Ascorbic acid was used as the reference standard. Data are presented as the mean ± standard deviation of three independent replicates. GraphPad Prism 10 was used to generate the graph, and a *p*-value of less than 0.05 was considered statistically significant.

Based on the EC50 values obtained from the DPPH assay, all tested compounds exhibited notable free radical scavenging activity. Ascorbic acid, used as the positive control, showed the lowest EC50 value, confirming its superior antioxidant potency. Among the organotin(IV) complexes, Complex 3 displayed the highest antioxidant activity, reflected in its lowest EC50 value, followed by Complexes 2 and 5, whereas Complexes 4 and 6 demonstrated moderate activity (Figure 8). Previous studies have also reported that organotin(IV) complexes with moderate antioxidant activity demonstrated increased anticancer potential via the modulation of oxidative stress and apoptotic signaling pathways [42]. Both vitamins also showed notable radical scavenging effects, with VD demonstrating comparatively higher activity, which is in line with previous studies [43].

The assessment served as an important indicator of their ability to counteract oxidative stress, which played a significant role in the development and progression of cancer. The observed antioxidant activity suggested that these compounds possessed the capability to neutralize harmful free radicals, thereby reducing cellular damage and potentially inhibiting cancerous growth.

#### 2.11.2. Anti-Inflammatory Assay

**Figure 9:**
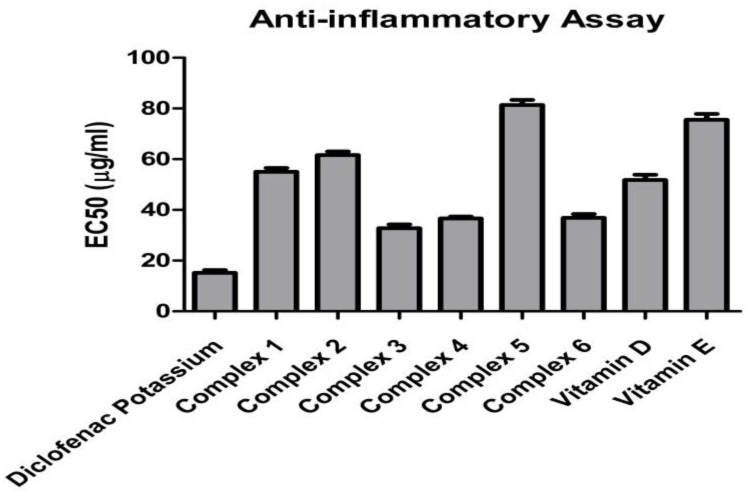
EC50 values representing the anti-inflammatory activity of organotin(IV) complexes and vitamins, determined using the protein denaturation inhibition assay. Diclofenac potassium was used as the reference standard drug. Data are expressed as mean ± standard deviation of three replicates. GraphPad Prism 10 was used to generate the graph, and a *p*-value of less than 0.05 was considered statistically significant.

Based on the EC50 values obtained from the protein denaturation inhibition assay, all tested compounds exhibited measurable anti-inflammatory activity when compared with diclofenac potassium, which served as the reference drug (Figure 9). Diclofenac potassium, as expected, showed the lowest EC50 value, reflecting its superior potency. Among the organotin(IV) complexes, Complex 3 displayed the strongest anti-inflammatory effect, evidenced by its lowest EC50 value among the complexes, followed by Complexes 4 and 6. The remaining organotin(IV) complexes exhibited moderate anti-inflammatory potential. Both vitamins also showed moderate inhibition relative to the reference drug, which is in line with previous studies [44,45].

The complexes demonstrated significant anti-inflammatory activity, suggesting their potential to mitigate inflammatory responses associated with BC and other related conditions. By modulating inflammatory pathways, the complexes have the potential to reduce tissue damage, inflammation-induced pain, and overall inflammatory burden.

#### 2.11.3. Cytotoxicity Assay and Cell Viability Assay

The cytotoxic potential of the synthesized organotin(IV) complexes, along with VD and VE, were evaluated in MCF-7 and MDA-MB-231 cell lines using MTT assays. The following formula was used to determine cell viability.

(1) $$Cell\;viability\;\left(\%\right)=\frac{\left((OD\;of\;cells\;treated\;with\;drug-OD\;of\;blank)\times 100\right)}{\left(OD\;of\;control\;cells-OD\;of\;blank\right)}$$

**Figure 10:**
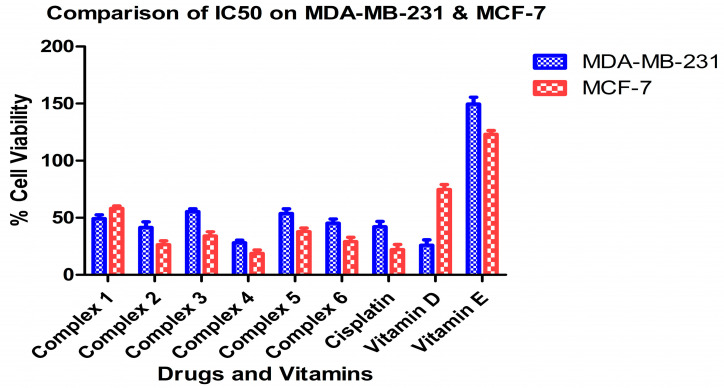
Comparison of IC50 values for organotin(IV) complexes and vitamins in MCF-7 and MDA-MB-231 cell lines. The IC50 values represent the concentration of each compound required to inhibit 50% of cell viability. Data are presented as the mean ± SEM based on three replicates, highlighting differential cytotoxic responses between the two cell lines. GraphPad Prism 10 was used to generate the graph, and a *p*-value of less than 0.05 was considered statistically significant.

The half-maximal inhibitory concentration (IC50) values were measured using GraphPad Prism software, and data are presented in Figure 10. According to our findings, VD showed values in the range of 10 to 40 µM for MDA-MB-231 and 30 µM to 100 µM for MCF7. VE showed IC_50_ values in the range of 50 µM–250 µM for both MDA-MB-231 and MCF-7. All organometallic complexes and the reference drug cisplatin showed IC_50_ in the range of 10 µM to 60 µM for both cell lines, indicating strong cytotoxic potential.

A comparative analysis among all the tested compounds revealed that Complex 4 exhibited most potent cytotoxic activity in both cell lines, surpassing the cytotoxic activity of cisplatin. Complex 2 also revealed significant activity specifically on MDA-MB-231, though to a lesser extent than Complex 4. Complexes 1 and 6 showed moderate activity, while Complexes 3 and 5 revealed weak cytotoxic effects. Both vitamins also exhibited significant cytotoxicity, which is in line with previous studies [46,47].

Our in silico docking and pharmacokinetic analyses identified Complex 4 as the most promising candidate, exhibiting favorable binding affinities, stability, and drug likeness parameters. These computational findings suggested that Complex 4 could possess a strong potential for biological activity and acceptable pharmacokinetic behavior. Subsequent in vitro validation through cytotoxicity assays provided compelling evidence in support of these predictions. Complex 4 demonstrated higher inhibitory effects against both MCF-7 (>1.16-fold) and MDA-MB-231 (>1.46-fold) BC cell lines, even surpassing the reference drug cisplatin in terms of efficacy. The enhanced performance of Complex 4 may be attributed to the presence of the chloro substituent, which is smaller and more electron-withdrawing and diffusive than the bulkier Bu or Ph groups. This combination likely increases the electrophilicity of the tin center and reduces steric hindrance, facilitating stronger interactions with biological targets and potentially improving cellular uptake. This concordance between computational and experimental results underscores the translational potential of Complex 4 as a lead compound for anticancer therapy. This is consistent with previous studies demonstrating strong cytotoxic and predictive in silico-in vitro association for organotin(IV) complexes and other organometallic complexes [13].

Complex 4 consistently outperformed the others, suggesting that the presence of the chloride ligand and the methyl substituents create an optimal balance of electronic effects, steric accessibility, and coordination behavior. These features likely enhance its reactivity and interaction with target substrates. Highlighting the superior performance of Complex 4 helps establish meaningful structure–activity relationships that are crucial for guiding the rational design of improved organotin complexes in the future.

Based on the biological effect of organotin(IV) derivatives, Complex 4 is likely to act through a multimodal mechanism. Organotin compounds mostly interact with DNA through groove binding or coordination with nucleobases, leading to replication stress and subsequent cell-cycle arrest [48]. In parallel, these complexes disrupt mitochondrial function and increase intracellular ROS, which together enhance mitochondrial membrane depolarization, cytochrome-c release, and caspase activation consistent with intrinsic apoptosis [13]. The strong cytotoxicity observed for Complex 4 therefore aligns with a combination of DNA interaction, ROS overproduction, loss of mitochondrial integrity, and apoptosis induction [49].

Building on these promising findings, Complex 4 was further evaluated for its combinatorial potential with VD and VE, both known for their strong anticancer and antioxidant properties, respectively [50,51]. The combined treatments revealed markedly higher cytotoxic effects compared to the individual agents alone. Notably, VD and VE enhanced the inhibitory effect of Complex 4 across all tested concentrations, indicating a clear synergistic interaction (Figures S33 and S34).

To quantify this interaction, the Chou–Talalay method was employed. The resulting combination index (CI) values ranged from 0.66 to 0.908 µM for MDA-MB-231 and 0.767–0.906 µM for MCF-7, all below 1, confirming synergy between Complex 4 and both vitamins. For combination treatments, Complex 4 and VD were used at a ratio of 1:3 in the MCF-7 cell line and 1:2.5 in the MDA-MB-231 cell line, while Complex 4 and VE were employed at a ratio of 1:6 in MCF-7 cells and 1:4 in MDA-MB-231 cells. These dose ratios were used to measure the corresponding CI values, indicating synergistic interactions in both cell lines. These findings suggest that the co-administration of Complex 4 with VD or VE could be an effective therapeutic strategy to enhance anticancer efficacy against BC cells. Previous studies have similarly highlighted the synergistic potential of organotin(IV) complexes when combined with bioactive or antioxidant molecules. Organotin derivatives are known to improve the bioavailability and cellular uptake of co-administered compounds, thereby enhancing therapeutic outcomes. Our in silico docking and pharmacokinetic analysis and pharmacokinetic identified Complex 4 as a potential candidate because of its favorable binding affinities, stability, and drug likeness parameters. Our experimental validation also provided compelling evidence of its superior biological inhibitory effects against both cell lines, even surpassing the refence drug cisplatin. This concordance among computational and in vitro findings suggests the strong translational potential of Complex 4 as a lead compound for further anticancer investigations. Based on this, Complex 4 was further assessed for its combinatorial potential with VD and VE against both cell lines. Our findings revealed that co-administration of VD or VE with Complex 4 resulted in significantly lower cell viability compared to when it was used alone. Interestingly, both vitamins enhanced the inhibitory effect of Complex 4, indicating a synergistic interaction (Figures S33 and S34).

**Figure 11:**
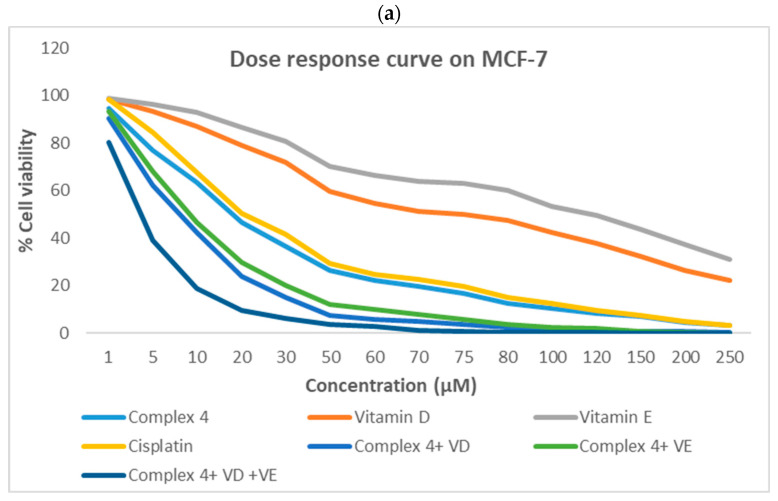

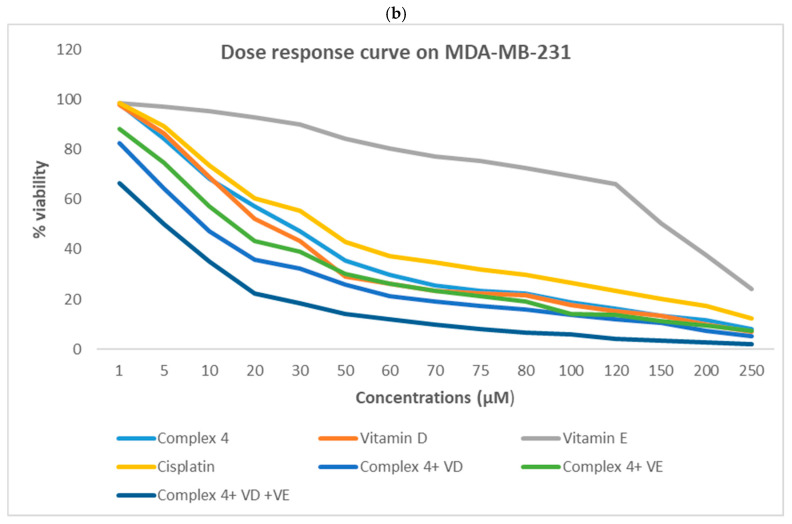
The dose–response effects of Complex 4, VD, VE, and their combinations were assessed in (**a**) MCF-7 and (**b**) MDA-MB-231 cell lines. Cisplatin was used as a reference drug. Cell viability was evaluated across a range of concentrations, and the resulting percentage viability was used to plot dose–response curves. Data are based on three independent replicates.

The dose-dependent effects of Complex 4, VD, and VE and their combinations were evaluated in MCF-7 and MDA-MB-231 cell lines. Cisplatin was used as a reference drug. Cell viability was assessed across a range of concentrations, and the resulting percentage cell viability was used to plot dose–response curves (Figure 11).

Overall, these findings suggest a therapeutic potential of combining Complex 4 with vitamins as a potential therapeutic strategy for increasing anticancer efficacy against BC cell lines, in line with previous findings [52,53]. For instance, organotin ethers containing thiamine (vitamin B1) have demonstrated potent anticancer effects, even outperforming cisplatin, while exhibiting lower systemic toxicity [54]. Likewise, triorganotin complexes combined with all-trans retinoic acid (vitamin A) suppressed the expression of key oncogenic proteins, including vimentin and annexin 5, in MDA-MB-231 cells [55]. Further, García et al. reported the development of mesoporous silica functionalized with folate and organotin(IV) complexes, which significantly enhanced anticancer efficacy through targeted delivery [56].

The incorporation of antioxidant molecules such as VE or VE-mimetics into metal complexes has also been shown to mitigate oxidative stress in non-cancerous cells and reduce the side effects of chemotherapy. Organotin(IV) complexes are well documented to induce cytotoxicity through excessive ROS generation, the disruption of mitochondrial membrane potential, and the activation of the intrinsic apoptotic pathway [57]. The combination of VD and VE most likely enhance mitochondrial redox homeostasis and apoptotic signaling pathways in ways that sensitize cells to organotin-induced stress [58]. VD modulates mitochondrial oxidative metabolism and transcription of redox and apoptosis-associated genes through VD-responsive elements. In many cancer models, VD increases oxidative phosphorylation, elevates basal ROS, and shifts the Bax/Bcl-2 ratio toward a pro-apoptotic state. These changes effectively lower the threshold for organotin-mediated mitochondrial permeability, transition, and cytochrome-c release [59]. VE also exerts antioxidant and redox-modulatory effects in tumor cells [60]. Some isoforms of VE (e.g., tocotrienols) promote lipid peroxidation, impair mitochondrial complex I activity, and reduce endogenous antioxidant capacity (e.g., SOD and GPx activity), thereby amplifying organotin-induced ROS accumulation and destabilizing mitochondrial membranes, facilitating earlier Δψm collapse and caspase-9/3 activation [61]. Thus, both vitamins converge mechanistically on the ROS–mitochondria–apoptosis axis, enhancing organotin-induced mitochondrial depolarization and apoptotic signaling. This provides a coherent biological rationale for the synergistic cytotoxicity and CI values observed. In addition to these effects, both VD and VE have been shown to inhibit NF-κB-driven transcription and attenuate PI3K/Akt signaling in several cancer models [62,63], leading to reduced anti-apoptotic signaling and a weakened cellular stress-response capacity [64]. Suppression of these pathways would lower the apoptotic threshold in the presence of organotin-induced mitochondrial damage and ROS accumulation. Therefore, the inhibition of NF-κB and PI3K/Akt provides an additional mechanistic explanation for the synergistic cytotoxicity observed between the vitamins and Complex 4. Russian researchers demonstrated that organotin derivatives containing 2,6-di-tert-butylphenol (a VE analog) displayed high radical scavenging activity and strong antimitotic potential via tubulin binding [65]. In another study, VE analogs integrated into organotin complexes effectively minimized the cytotoxic impact on healthy cells while maintaining potent antitumor efficacy [66]. These studies are consistent with our results, suggesting that combining organotin(IV) complexes with VD and VE not only enhances anticancer potency but may also aid in mitigating some of the limitations associated with conventional chemotherapy.

Overall, the findings of our study suggest that the combination of organotin(IV) complexes with VD and VE could represent a novel and highly effective therapeutic strategy for BC. The synergistic effects observed in both MDA-MB-231 and MCF-7 cell lines indicate that this combination not only enhances the antitumor activity of each component but also holds the potential to overcome the limitations of traditional chemotherapy, such as side effects and drug resistance.

## 3. Materials and Methods

### 3.1. Drugs and Reagents

VD (Cholecalciferol) and VE (DL-α-Tocopherol) were purchased from Solarbio (Beijing Solarbio Science & Technology Co., Ltd., Beijing, China) and MACKLIN (Shanghai Macklin Biochemical Co., Ltd., Shanghai, China), respectively. BC cell lines MCF-7 (HTB-22™) and MDA-MB-231 (HTB-26™) were purchased from the American Type Culture Collection (ATCC, Rockville, Manassas, MD, USA). Reagents used for cell cultures, such as RPMI 1640 culture medium, Fetal Bovine Serum (FBS), antibiotics (Penicillin-Streptomycin), Trypsin-EDTA solution, and Nuclease-Free water were purchased from ThermoFisher Scientific (GibcoTM, ThermoFisher Scientific, Waltham, MA, USA). Phosphate-buffered saline (PBS), MTT dye, cisplatin, trypan blue solution, carbon disulphide (CS_2_), and organotin(IV) chlorides were purchased from Sigma-Aldrich (Sigma Aldrich, Rockville, MD, USA). Various solvents such as chloroform, methanol, toluene, n-hexane, ethanol, acetone, and DMSO of analytical grade were purchased from Merck, Darmstadt, Germany.

### 3.2. Equipment Used for Elemental Analyses

The elemental analyses were conducted using a LECO CHNS-932 analyzer. Melting points were determined utilizing an electrothermal melting point apparatus (MP-D Mitamura Riken Kogyo, Tokushima, Japan). FT-IR spectra were measured as KBr discs employing a Perkin Elmer spectrum 1000 (Shelton, CT, USA) within the range of 4000–250 cm^−1^. The UV–Visible absorption spectra were acquired employing a 1601 double-beam (Shimadzu, Kyoto, Japan) spectrophotometer, Japan. The setup included a deuterium lamp and a 50 W halogen lamp as the light source. Measurements were conducted using quartz cells with a path length of 1 cm. ^1^H- and ^13^C-NMR were recorded on a Bruker AC 300 MHFT-NMR (Karlsruhe, Germany) in chloroform. ^119^Sn-NMR was recorded on Avance 400 MHz TXO 10 mm XWINNMR (Rheinstetten, Germany), where Me_4_Sn was used as a standard reference. The coupling pattern of each signal in ^1^H-NMR is given with chemical shift (s = singlet; d = doublet; t = triplet; m = multiplet). All *^n^J*[^119^Sn–^1^H] and [^119^Sn–^13^C] coupling constants are reported in square brackets, while ^1^H–^1^H coupling constants are given in small brackets.

### 3.3. Synthesis of Sodium(E)-4-Styryl Piperazine-1-Carbodithioate

The sodium salt of the ligand was synthesized following a previously reported procedure [67]. Specifically, a mixture comprising 2 g of (E)-1-styrylpiperazine (0.0106 moles), 0.43 g of sodium hydroxide (0.0106 moles), and 0.7 mL of carbon disulfide (0.0116 moles) was combined with 50 mL of methanol. A yellow solid product was produced by stirring the mixture for an hour at 0 °C. It was then cleaned with methanol and allowed to air-dry.

Yield: 95%, Melting Point: 100–103 °C, Elemental analysis, % found (calculated); C_13_H_15_N_2_NaS_2_: C, 53.45 (54.52); H, 5.23 (5.28); N, 9.63 (9.78), UV–Vis (DMSO) λ_max_/nm: 259. FT-IR (cm^−1^): 1065 ν(C-S), 1463 ν(C-N), ^1^H-NMR (ppm): Ar-H [6.95–6.99 (m), 6.82–6.84 (m), 7.45–7.48 (m)], CH=CH [7.56 (d, 12), 5.24 (d, 12)], Piperazine-H [2.94–2.97 (t, 9.3), 2.19–2.22 (t, 9.0)], ^13^C-NMR (ppm): 209.8 (C=S), 138.4, 131.4, 126.1 (Ar-C) 105.9, 116.8. (CH=CH), 49.8, 52.3 (Piperazine-C) (see spectra in Supplementary Materials).

### 3.4. General Procedure for the Synthesis of Complexes

The preparation of the complexes followed our group’s established method [15,68]. Generally, the reaction involved triorganotin chloride and diorganotin(IV) dichlorides with a ligand salt in dry toluene, maintaining a 1:1 molar ratio, yielding tri- and di-alkylstannyl (E)-4-styrylpiperazine-1-carbodithioates, respectively. Meanwhile, dimethyltin(IV) dichloride, in a 1:2 molar ratio with the ligand salt, produced dimethylstannyl bis-[(E)-4-styrylpiperazine-1-carbodithioate]. The resulting mixture underwent reflux for 7–8 h with continuous stirring, followed by NaCl removal through filtration. Subsequently, the solvent was evaporated using a rotary evaporator to obtain the desired product, later recrystallized from a methanol–chloroform mixture. The procedure and numbering pattern for the alkyl or aryl groups directly attached to the tin center are shown in Scheme 1 to interpret the NMR spectra.

### 3.5. Synthesis of Trimethylstannyl (E)-4-Styrylpiperazine-1-Carbodithioate (1)

Yield: 0.33 g, 79%, Melting Point: 44–45 °C, Elemental Analysis, % found (calculated): C_16_H_24_N_2_S_2_Sn: C, 44.85 (44.98); H, 5.63 (5.66); N, 6.52 (6.56), UV–Vis (DMSO) λ_max_/nm: 257, FT-IR (cm^−1^): 992 ν(CS_2_)_sym_, 1131 ν(CS_2_)_asym_, (Δν = 139), 1474 ν(C-N), 352 ν(Sn-S), 544 ν(Sn-C), ^1^H-NMR (ppm): Ar-H [6.86–6.99 (m), 7.11–7.14 (m)], CH=CH [7.53 (d, 12.3), 5.54 (d, 12.3)], Piperazine-H [2.98, (t, 9.6), 3.10 (t, 9.6)], Hα [0.69 (s, [*^n^J*(^119^Sn–^1^H= 57), θ = 109.6°], ^13^C-NMR (ppm): C=S (198.2), Ar-C (138.2, 131.4, 126.0, 125.73), CH=CH (116.9, 100.4), piperazine-C (51.6, 52.1), H_3_C-Sn (1.04) (see spectra in Supplementary Materials).

### 3.6. Synthesis of Tributylstannyl (E)-4-Styrylpiperazine-1-Carbodithioate (2)

Yield: 0.91 g, 82%, Liquid, Elemental analysis, % found (calculated); C_25_H_42_N_2_S_2_Sn: C, 54.31 (54.25); H, 7.61 (7.65); N, 5.10 (5.06), UV–Vis (DMSO) λ_max_/nm: 256, FT-IR (cm^−1^): 994 ν(CS_2_)_sym_, 1140 ν(CS_2_)_asym_, (Δν = 146), 1469 ν(C-N), 349 ν(Sn-S), 539 ν(Sn-C), ^1^H-NMR (ppm): Ar-H [6.89–6.98 (m), 7.06–7.08 (m), 7.11–7.13 (m)], CH=CH [(5.39 (d, 12.3), 7.58 (d, 12.3)], Piperazine-H [2.29 (t, 9.6), 2.97 (t, 9.6)] Hα,β [1.61–1.72 (m)], Hγ [1.30–1.45 (m)], Hδ [0.95 (t, 7.8)], ^13^C-NMR (ppm): C=S (198.9), Ar-C (140.4, 138.2, 131.4, 125.9), CH=CH (116.8, 101.3), piperazine-C (51.7, 52.2), C-α (17.8) [*^2^J*(^119^Sn–^13^C) = 349.16], C-β (28.9) [*^2^J*(^119^Sn–^13^C = 20.95] C-γ (27.2) [*^2^J*(^119^Sn–^13^C = 68.03)], C-δ (13.9). ^119^Sn-NMR (ppm): 29.7 (see spectra in Supplementary Materials).

### 3.7. Synthesis of Triphenylstannyl (E)-4-Styrylpiperazine-1-Carbodithioate (3)

Yield: 0.93 g, 75.4%, Melting Point: 113–115 °C, Elemental Analysis, % found (calculated); C_31_H_30_N_2_S_2_Sn: C, 60.55 (60.70); H, 4.98 (4.93); N, 4.57 (4.57), UV–Vis (DMSO) λ_max_/nm: 254, FT-IR (cm^−1^): 991 ν(CS_2_)_sym_, 1128 ν(CS_2_)_asym_ (Δν = 137), 1473 ν(C-N), 357 ν(Sn-S), 549 ν(Sn-C), ^1^H-NMR (ppm): Ar-H [6.88–6.98 (m), 7.09–7.14 (m), 7.34–7.39 (m)], CH=CH [(5.27 (d, 12.3), 7.50 (d, 12.3)], Piperazine-H [2.92 (t, 9.6), 3.13 (t, 9.6), Hα,β [7.40–7.46 (m)], Hγ [7.75–7.77 (m), Hγ [7.78–7.79 (m)], ^13^C-NMR (ppm): C=S (196.4), Ar-C (140.2, 138.2, 128.2, 125.8), CH=CH (116.7, 106.3), piperazine-C (51.5, 52.8), C-α (142.2), C-β (136.7), C-γ (128.5), C-δ (133.7). ^119^Sn-NMR (ppm): 58.8 (see spectra in Supplementary Materials).

### 3.8. Synthesis of Chlorodimethylstannyl (E)-4-Styrylpiperazine-1-Carbodithioate (4)

Yield: 0.62 g, 67.8%, Melting Point: 123–127 °C, Elemental Analysis, % found (calculated); C_28_H_36_S_4_Sn: C, 40.17 (40.25); H, 4.68 (4.73); N, 6.22 (6.26), UV–Vis (DMSO) λ_max_/nm: 252, FT-IR (cm^−1^): 995 ν(CS_2_)_sym_, 1126 ν(CS_2_)_asym_, (Δν = 131), 1480 ν(C-N), 354 ν(Sn-S), 535 ν(Sn-C), 315 ν(Sn-Cl), ^1^H-NMR (ppm): Ar-H [6.90–6.92 (m), 6.98–7.00 (m), 7.09–7.14 (m)], CH=CH [(7.53 (d, 12.3), 5.54 (d, 12.3)], Piperazine-H [2.27, (t, 9.6), 3.03 (t, 9.6), Hα (1.29), ^13^C-NMR (ppm): C=S (196.6), Ar-C (139.8, 138.4, 131.4, 126.0), CH=CH (116.7, 101.2), piperazine-C (51.4, 52.2), H_3_C-Sn (10.06) (see spectra in Supplementary Materials).

### 3.9. Synthesis of Chlorodibutylstannyl (E)-4-Styrylpiperazine-1-Carbodithioate (5)

Yield: 0.77 g, 71.1%, Melting Point: 91–94 °C, Elemental Analysis, % found (calculated); C_21_H_33_ClN_2_S_2_Sn: C, 47.42 (47.43); H, 6.27 (6.25); N, 5.24 (5.27), UV–Vis (DMSO) λ_max_/nm: 251, FT-IR (cm^−1^): 992 ν(CS_2_)_sym_ 1139 ν(CS_2_)_asym_, (Δν = 147), 1485 ν(C-N), 360 ν(Sn-S), 540 ν(Sn-C), 319 ν(Sn-Cl), ^1^H-NMR (ppm): Ar-H [6.89–6.98 (m), 7.07–7.09 (m), 7.12–7.14 (m)], CH=CH [(5.16 (d, 12.3), 7.55 (d, 12.3)], Piperazine-H [2.29 (t, 9.6), 3.02 (t, 9.6)] Hα,β,γ [1.28–1.53 (m)], Hδ [0.98 (t, 15)], ^13^C-NMR (ppm): C=S (197.3), Ar-C (139.8, 138.4, 126.1, 125.9), CH=CH (116.9, 101.7), piperazine-C (51.4, 52.2), C-α (20.6), C-β (27.8) [*^2^J*(^119^Sn–^13^C)= 33.63] C-γ (29.4), C-δ (13.7). ^119^Sn-NMR (ppm): −144.5 (see spectra in Supplementary Materials).

### 3.10. Synthesis of Dimethylstannyl Bis-(E)-4-Styrylpiperazine-1-Carbodithioate (6)

Yield: 1.00 g, 73.2%, Melting Point: 65–67 °C, Elemental Analysis, % found (calculated); C_28_H_36_N_4_S_4_Sn: C, 49.70 (49.78); H, 5.32 (5.37); N, 8.25 (8.29), UV–Vis (DMSO) λ_max_/nm: 254, FT-IR (cm^−1^): 983 ν(CS_2_)_sym_, 1118 ν(CS_2_)_asym_, (Δν = 135), 1487 ν(C-N), 351 ν(Sn-S), 533 ν(Sn-C), ^1^H-NMR (ppm): Ar-H [6.86–6.99 (m), 7.05–7.08 (m), 7.12–7.14 (m)], CH=CH [(5.25 (d, 12.3), 7.56 (d, 12.3)], Piperazine-H [2.96–2.99 (t, 9.6), 3.01–3.04 (t, 9.9)] 0.84 (s, Hα, [60], θ = 111.8°). ^13^C-NMR (ppm): C=S (198.2), Ar-C (137.8, 127.8, 126.8, 125.1), CH=CH (115.3, 103.9), piperazine-C (51.5, 52.1), Sn-CH_3_ (21.5) (see spectra in Supplementary Materials).

### 3.11. In Silico Analysis

#### 3.11.1. Data Collection

Transcriptome analysis data for BC was obtained from ‘The Human Protein Atlas database’ was accessed on 15 July 2022 using link (https://www.proteinatlas.org/) [69]. The analysis report revealed that 72% (14,418/20,090) of the total screened protein exhibited expression in BC. To prioritize targets, the GEPIA web server used for differential expression analysis was accessed on 15 July 2022 using link (http://gepia.cancer-pku.cn/), which integrates RNA-seq data from the cancer genome atlas (TCGA) (tumor samples) and genotype-tissue expression (GTEx) (normal tissues). One-way ANOVA was applied for differential gene expression analysis, with log_2_(TPM + 1)-transformed expression data, |log_2_FC| ≥ 1, and a q-value cutoff of 0.01. Overall survival (OS) and disease-free survival (DFS) analyses were carried in GEPIA using the log-rank test based on the Cox-PH model and 95% confidence interval. The gene expression threshold of 50% median value was set to split the high and low expression cohorts. Genes exhibiting differential expression and prognostic value were selected for further analysis.

#### 3.11.2. Protein–Protein Interaction (PPI) Analysis

Protein–protein interactions among the shortlisted proteins were evaluated using the STRING database version 12.0, accessed on 5 August 2022 using link (https://string-db.org/), focusing on Homo sapiens; the search parameters were set to include interactions with a high confidence score of 0.7 [70]. The interaction types among the key proteins were illustrated through network edges, representing both experimentally validated interactions and predicted gene associations.

### 3.12. Molecular Docking

#### 3.12.1. Data Source

The PDB structures of the proteins were obtained from the Protein Data Bank accessed on 5 Aug 2022 through https://www.rcsb.org/. The PDB IDs of proteins were IL-22 (PDB ID: 3DGC), NFκB (PDB ID: 1A3Q), Akt (PDB ID: 3CQW), CXCR4 (PBD: 3ODU), ER-alpha (PDB ID: 2JF9), and STAT (PDB ID: 1YVL). The structure of ligands was taken from PubChem (https://pubchem.ncbi.nlm.nih.gov/). The ligands were VD (CID_5280795), VE (CID_14985), and organotin(IV) complexes.

#### 3.12.2. Ligand Preparation

Vitamins and organotin(IV) complexes were prepared as ligands following the established protocol [71]. The 2D structures of the organotin(IV) complexes were generated in ChemDraw version 23.0 and converted into 3D structures using Molecular Operating Environment (MOE) software version 2022.02 [72]. These structures were subsequently protonated at pH 7.4, and the addition of hydrogen atom, partial charge assignment, and energy minimization were carried using the MMFF94x force field in MOE [73]. Additional energy minimization was carried out with Biovia Discovery Studio (BDS) and AutoDock Tools 1.5.7 [74].

#### 3.12.3. Receptor/Protein Preparation

The 3D crystal structures of target proteins were retrieved from the RCSB PDB database and prepared for docking. Preprocessing included energy minimization, the addition of polar hydrogens, the assignment of Kollman charges, and the removal of water molecules, irrelevant ligands, and ions using AutoDock Tools 1.5.7 [71]. Further refinements were carried though MOE software [75].

#### 3.12.4. Proteins Molecular Docking Analysis

AutoDock 1.5.7 and AutoDock Vina

Molecular docking was performed using AutoDock 1.5.7 (The Scripps Research Institute, La Jolla, CA, USA) [71]. Binding pockets were defined using AutoGrid with a grid spacing of 0.375 Å and grid dimensions of 40 × 40 × 40 along the X, Y, and Z axes. Docking simulations were carried out with the Lamarckian genetic algorithm (10 runs), treating macromolecules as rigid and ligands as flexible [76]. Binding affinity was evaluated based on the lowest binding energy values.

#### 3.12.5. Molecular Operating Environment (MOE)

Molecular docking of the ligands with the selected proteins was performed using MOE. Binding pockets were identified with the Site Finder tool. Proteins were preprocessed by protonation at pH 7.4, energy minimization with the Amber10 force field, and hydrogen/charge assignment [77]. Ligands were treated as flexible, and docking was carried out using the induced-fit protocol with 10 poses per ligand, the Alpha Triangle placement method [78], and London dG scoring. The lowest binding energy was considered the most favorable.

#### 3.12.6. Interaction Analysis

Protein–ligand interactions were visualized using protein–ligand interaction profiler (PLIP) [79] and BDS [80], which identified hydrogen bonds, hydrophobic contacts, π–π interactions, π–sulfur bonds, and other non-covalent interactions.

#### 3.12.7. Molecular Docking Simulations

Protein flexibility of the top-ranked ligand–protein complexes was assessed using the CABS-flex 2.0 server (http://biocomp.chem.uw.edu.pl/CABSflex2, accessed on 23 October 2025). Simulations were run with default parameters (50 cycles, 10 ns), and flexibility was evaluated by root mean square fluctuation (RMSF). Flexibility simulations generated by this server have shown a strong correlation with NMR results [81].

#### 3.12.8. Pharmacokinetic Analysis

Pharmacokinetic and drug likeness properties of organotin complexes and vitamins were predicted using SwissADME version 2.6.0 (http://www.swissadme.ch/), based on their ADMET properties [71]. Toxicity, bioactivity, and physicochemical parameters were evaluated with Molinspiration (http://www.molinspiration.com/) [82], while potential acute and organ-specific toxicities were predicted using ProTox-II (http://tox.charite.de/protox_II/, accessed on 23 October 2025) [83].

### 3.13. In Vitro Analysis

#### 3.13.1. Antioxidant Assay

The antioxidant activity of vitamins and organotin(IV) complexes was assessed using the DPPH radical scavenging assay as described previously [84], with ascorbic acid as standard. Briefly, each sample was tested across a concentration range of 10–100 µg/mL, samples were incubated with 0.1 mM DPPH in methanol for 30 min in the dark, and absorbance was measured at 517 nm. Percentage inhibition was calculated relative to control. Statistical analysis was performed using one-way ANOVA followed by Tukey’s post hoc test using GraphPad Prism 10, with *p* < 0.05 considered statistically significant.

#### 3.13.2. Anti-Inflammatory Assay

Anti-inflammatory activity was evaluated using the protein denaturation assay as described previously [85], with diclofenac potassium as standard. Briefly, each sample was tested across a concentration range of 10–100 µg/mL; samples were incubated with BSA, heated at 72 °C, and cooled, and absorbance was measured at 660 nm. The percentage inhibition of protein denaturation was calculated relative to control. Statistical analysis was performed using one-way ANOVA followed by Tukey’s post hoc test using GraphPad Prism 10, with *p* < 0.05 considered statistically significant.

#### 3.13.3. Cell Culture

MCF-7 and MDA-MB-231 cell lines were cultured in RPMI-1640 medium supplemented with 10% FBS and 2% penicillin-streptomycin, under standard conditions at 37 °C in 5% CO_2_ and 95% humidified atmosphere. Cells were passaged at 75–80% confluency using trypsin-EDTA, with medium refreshed every 3–4 days for MCF-7 and 2–3 days for MDA-MB-231. Experiments were conducted using cells between passages 3 to 5.

#### 3.13.4. MTT Assay

Cell proliferation and viability were evaluated by the MTT assay [79]. Cells were seeded at 10,000 cells/well and treated for 24 h with varying concentrations of VD (10–80 µM), VE (50–400 µM), the complexes (10–80 µM), or cisplatin. After incubation with MTT (5 mg/mL) for ~4 h, formazan crystals were dissolved in DMSO and absorbance recorded at 550 nm. IC_50_ values were calculated, and drug interactions were assessed using the Chou–Talalay method to determine the combination index (CI) [86].

#### 3.13.5. Statistical Analysis

All in vitro experiments were performed in triplicate, and data are presented as mean ± SEM. Responses were normalized to control values. Statistical significance was determined using a two-tailed Student’s *t*-test, with *p* < 0.05 considered statistically significant. Analyses were performed using GraphPad Prism 10.

## 4. Conclusions

The findings of this study provide compelling evidence for the therapeutic potential of combining organotin(IV) dithiocarbamate complexes with VD and VE in BC treatment. The synergistic effects observed between these synthetic complexes and vitamins suggest a promising strategy to overcome the limitations of current BC therapies. Specifically, Complex 4, in combination with VD and VE, demonstrated superior anticancer activity compared to individual treatments, exhibiting potent inhibition of cell proliferation, apoptosis induction, and antioxidant properties. This approach not only enhances the bioavailability and cellular uptake of the compounds but also presents a novel avenue for BC therapy by integrating synthetic and natural agents. The results contribute to the growing body of research supporting the use of combination therapies in cancer treatment, offering a foundation for future exploration of this promising strategy.

### Limitations and Future Directions

This study’s limitations include its in vitro nature, which does not fully capture the complexity of cancer biology, requiring further validation in animal models to assess in vivo efficacy and safety. The focus on two BC cell lines limits the generalizability of the results, and additional testing on other cell lines is needed. Moreover, the long-term toxicity and pharmacokinetics of the organotin complexes in combination with VD and VE remain unexplored. Because ADME and toxicity prediction platforms are less reliable for metal-based complexes, the in silico results presented in this study are preliminary and will be complemented by experimental validation in future work. Future research should focus on in vivo validation of the organotin(IV) complexes combined with VD and VE, evaluating their effects on different BC subtypes. Long-term toxicity studies and pharmacokinetic assessments are necessary to assess clinical feasibility. Optimizing dosing and delivery methods could further enhance therapeutic efficacy in BC treatment.

## Data Availability

The original contributions presented in this study are included in the article/Supplementary Materials. Further inquiries can be directed to the corresponding author.

## Supplementary Materials

The following supporting information can be downloaded at: https://www.mdpi.com/article/10.3390/ph19040571/s1.
