# Supplementary material for: Synergistic Potential of Organotin(IV) Carbodithioate Derivatives with Vitamins D and E in MCF-7 and MDA-MB-231 Breast Cancer Cells
Source: Pharmaceuticals (Basel). 2026 Apr 2;19(4):571. doi: 10.3390/ph19040571 (PMC13119188; doi:10.3390/ph19040571)
Supplement: Supplementary file 1 [file pharmaceuticals-19-00571-s001.zip › pharmaceuticals-3975791-supplementary.pdf]

Supplementary File

Synergistic Potential of Organotin(IV) Carbodithioate Derivatives with Vitamins D and E in MCF-7 and MDA-MB-231 Breast Cancer Cells

Table S1: Bindings affinities and molecular interaction profiles of organotin (IV) complexes (1-6) and both vitamins D and E with targeted breast cancer related proteins (a) Akt, (b) CXCR4, (c) ER alpha, (d) IL-22, (e) NF-κB, and (f) STAT1. The table summarizes binding energies, interacting ligand atoms, ligand–protein contact residues, and the nature of molecular interactions.

(a)

Akt	Scores	Ligand atoms	Protein residues	Interactions	Distance
**Complex 1**	-5.72622	2611	LYS-297	Hydrophobic	3.69
			MET-392	Hydrogen bonding	3.25
			MET-392	C-H bond	2.69
			GLU-1157	C-H bond	2.60
			GLU-1157	C-H bond	2.94
			LYS-1161	Π-alkyl	4.32
			HIS-1158	Π-alkyl	4.81
**Complex 2**	-7.04561	2632, 2636, 2610, 2617	PHE-161<br>HIS-194<br>LEU-295<br>LEU-295	Hydrophobic	3.72<br>3.59<br>2.71<br>3.69
			Glu-394	H-Bonding	2.36
			Glu-393	Attractive charge	3.96
			Glu-393	C-H bond	2.65
**Complex 3**	-6.9241	2638, 2637, 2642, 2625	PHE-161<br>PHE-161<br>HIS-194<br>LEU-295	Hydrophobic	2.84<br>2.24<br>3.29<br>3.37
		2621, 2622, 2623, 2624, 2625	HIS-194	Π-Stacking	3.86
			HIS-1158	Π-Sulphur	4.18

			LYS-1161	Π-alkyl	4.20
			GLU-394	H-bonding	2.15
**Complex 4**	-5.74177	2627, 2626	HIS-194<br>GLU-198	Hydrophobic	3.19<br>3.99
			GLU-1157	C-H bond	2.67
			GLU-1157	C-H bond	2.75
			MET-392	C-H bond	2.89
			MET-1154	Alkyl	4.81
			GLU-394	Attractive charge	4.67
**Complex 5**	-6.58214	2608, 2609, 2610, 2632, 2633, 2634	LYS-297	Π-Cation	3.36
			TYR-356	C-H bond	2.96
			GLU-394	C-H bond	2.62
			MET-392	C-H bond	2.71
			HIS-1158	Π-Stacking	4.6
**Complex 6**	-7.74209	2627, 2626	PHE-161<br>HIS-194	Hydrophobic	3.80<br>3.62
			GLU-1157	Attractive charge	3.51
			GLU-1157	Attractive charge	3.58
			GLU-394	Attractive charge	4.03
			GLU-394	Attractive charge	4.81
			MET-392	C-H bond	2.44
			LYS-350	C-H bond	2.46
**Vitamin D**	-8.3	3283, 3288, 3286, 3287, 3281, 3305, 3305, 3306	PHE-161<br>PHE-161<br>LYS-179<br>LEU-181<br>HIS-194<br>PHE-236<br>GLU-278<br>PHE-442	Hydrophobic	3.94<br>3.48<br>3.76<br>3.68<br>3.83<br>3.66<br>3.69<br>3.81
			PHE-442	Π-alkyl	4.67
**Vitamin E**	-8.6	3291, 3290, 3283, 3301,	LEU-156<br>LEU-156	Hydrophobic	3.75<br>3.66

		3299	VAL-164<br>LEU-181<br>LEU-181		3.46<br>3.97<br>3.61
			Val-164	Π-sigma	3.85

(b)

CXCR4	Scores	Ligand atoms	Protein residues	Interactions	Distance
**Complex 1**	-6.28407		HIS-113	π-Sulphur	4.74
			ASP-187	C-H bond	2.74
			ASP-187	C-H bond	2.68
			ASP-187	Attractive charge	4.74
**Complex 2**	-6.92816	14977, 14980, 14977, 14954	TRP-94<br>TRP-94<br>ASP-97<br>ILE-284	Hydrophobic	3.49<br>3.83<br>3.54<br>3.37
			SER-285	C-H bond	2.57
			HIS-281	Π-alkyl	4.03
			ASP-97	Attractive charge	4.83
**Complex 3**	-6.71557	14984, 14966, 14959, 14961, 14956, 14955, 14983, 14956	TRP-125<br>LEU-132<br>LEU-151<br>LEU-151<br>VAL-155<br>VAL-158<br>ILE-213<br>ILE-221	Hydrophobic	3.81<br>3.57<br>3.70<br>3.90<br>3.09<br>3.59<br>3.78<br>3.18
		14964, 14965, 14966, 14967, 14968, 14969	TYR-135	Π-Stacking	5.16
			LEU-151	Π-Sigma	2.56
			VAL-155	Π-alkyl	4.63
			CYS-218	Π-alkyl	5.26
**Complex 4**	-7.00082	14965, 14867, 14969	ILE-259<br>ILE-284<br>ILE-284	Hydrophobic	3.65<br>3.74<br>3.70
			HIS-113	H-bonding	2.55
			ASP-187	Attractive charge	4.21
			HIS-113	π-Sulphur	4.27
			GLU-288	C-H bond	2.49
			TRP-94	Π-alkyl	4.57
**Complex 5**	-7.43394	14971, 14972<br>14973, 14971<br>14971, 14977<br>14976	TRP-94<br>TRP-94<br>ASP-97<br>TRP-102	Hydrophobic	3.42<br>3.70<br>3.78<br>3.56

			VAL-112<br>VAL-196<br>LEU-266		3.81<br>3.59<br>3.90
			ASP-262	π-Sulphur	3.46
			SER-285	Hydrogen bonding	3.39
			HIS-113	π-Anion	4.77
			GLU-288	C-H bonding	2.96
**Complex 6**	-8.1024	14953, 14951, 14964, 14984, 14985	ARG-30<br>GLU-31<br>VAL-196<br>HIS-281<br>LYS-282	Hydrophobic	3.98<br>3.84<br>3.60<br>3.41<br>3.61
			ARG-188	H-bonding	3.09
			ASP-187	Sulphur-X	3.13
			GLN-200	C-H bond	2.50
			HIS-281	C-H bond	2.97
			CYS-28	C-H bond	3.10
			ASN-278	C-H bond	2.80
			ASP-187	Attractive charge	4.00
**Vitamin D**	-8.0	9082, 9083, 9082, 9063, 9063, 9069, 9062, 9063	ILE-138<br>ILE-138<br>LYS-234<br>GLU-1005<br>ILE-1009<br>TRP-1158<br>TRP-1158<br>TYR-1161	Hydrophobic	3.81<br>3.35<br>3.65<br>3.66<br>3.43<br>3.30<br>3.98<br>3.59
			LYS-234	Alkyl	3.83
			HIS-1006	H-bonding	3.03
**Vitamin E**	-7.9	9067, 9081, 9086, 9089, 9085, 9072, 9082, 9083	TRP-125<br>TRP-125<br>LEU-167<br>LEU-167<br>VAL-206<br>LEU-210<br>LEU-210<br>ILE-213	Hydrophobic	3.53<br>3.92<br>3.72<br>3.98<br>3.68<br>3.62<br>3.68<br>3.64
		1318 (acceptor) 9090 (Donor)	GLY-159	Hydrogen bonding	2.77
			TRP-125	Π-Π Stacking	3.83
			ILE-213	Π-alkyl	3.96

(c)

ER alpha	Scores	Ligand atoms	Protein residues	Interactions	Distance
**Complex 1**	-5.92615	10776, 10777, 10777,10772, 10776	LEU-384<br>LEU-391<br>PHE-404<br>ILE-424<br>LEU-428	Hydrophobic	3.83<br>3.39<br>3.42<br>3.25<br>3.91
			THR-347	H-bonding	3.24
			LEU-346	C-H bonding	2.35
			TRP-383	π-Sulphur	5.10
			LEU-525	π-Alkyl	4.69
**Complex 2**	-7.72863		SER-1<br>THR-347	C-H bonding	2.39<br>2.24
			ALA-350	π-Alkyl	4.12
**Complex 3**	-2.32003	10787, 10777, 10806, 10779, 10804, 10805, 10804, 10800, 10805	LEU-320<br>GLU-323<br>PRO-324<br>ILE-326<br>MET-357<br>TRP-360<br>ILE-386<br>LEU-387<br>LYS-449	Hydrophobic	3.60<br>3.43<br>2.82<br>3.95<br>3.90<br>3.99<br>3.05<br>3.75<br>3.75
			TRP-393	H-bonding	2.33
			PHE-445	π-Sulphur	3.97
			ILE-326	π-Alkyl	4.64
**Complex 4**	-5.27469	10790, 10791, 10785	LEU-346<br>LEU-346<br>LEU-384	Hydrophobic	3.75<br>3.70<br>3.15
			SER	H-bonding	2.52
			THR-347	C-H bonding	2.89
			LEU-525	Alkyl	4.61
**Complex 5**	-7.69538	10793, 10792, 10788, 10774	LEU-387<br>PHE-404<br>ILE-424<br>LEU-525	Hydrophobic	3.63<br>3.49<br>3.60<br>3.61
			TRP-383	π-Sulphur	4.99
			THR-347	C-H bonding	2.68
			THR-347		2.78
			LEU-391	π-alkyl	4.32
			MET-421		4.04
**Complex 6**	-2.3671	10789	ILE-424	Hydrophobic	3.57
			LEU-525	C-H bonding	2.39
			MET-522	π-Alkyl	3.92
			TRP-383	π-Sulphur	4.93
**Vitamin D**	-6.9	6538, 6526,	TYR-328<br>ARG-394	Hydrophobic	3.69<br>3.55

		6530, 6523, 6536	GLU-397<br>PRO-397<br>PRO-406		3.86<br>3.51<br>3.80
			LEU-440	H-bonding	2.53
			ILE-326	π-Alkyl	4.06
			ARG-394	π-Alkyl	4.12
**Vitamin E**	-8.2	6530, 6538, 6512, 6525, 6514, 6514, 6522, 6525, 6533, 6536, 6538, 6539, 6538, 6537, 6524	LEU-346<br>LEU-346<br>THR-347<br>ALA-350<br>LEU-354<br>TRP-383<br>TRP-383<br>TRP-383<br>LEU-387<br>LEU-391<br>PHE-404<br>ILE-424<br>PHE-425<br>LEU-428<br>LEU-525	Hydrophobic	3.50<br>3.62<br>3.53<br>3.40<br>3.99<br>3.65<br>3.68<br>3.56<br>3.95<br>3.74<br>3.65<br>3.58<br>3.56<br>3.78<br>3.25
			LEU-525	π-Sigma	3.94
			ALA-350	π-Alkyl	3.91
			ALA-350	π-Alkyl	3.76
			LEU-525	π-Alkyl	4.19

(d)

**IL-22**	**Scores**	**Ligand atoms**	**Protein residues**	**Interactions**	**Distance**
**Complex 1**	-5.63338	11093, 11095, 11095	LEU-116<br>MET-172<br>TRP-208	Hydrophobic	3.79<br>3.90<br>3.59
			GLN-89	C-H bonding	2.59
**Complex 2**	-6.7682	11098, 11096, 11119	LEU-88<br>LEU-116<br>ILE-179	Hydrophobic	3.90<br>3.51<br>3.43
			ASN-176	C-H bonding	2.36
			ASP-162	C-H bonding	2.37
			HIS-161	π-Sulphur	5.55
			ILE-159	Attractive charge	4.58
			LEU-88	Attractive charge	4.90
**Complex 3**	-6.55965	11108, 11107, 11124, 11103,	LYS-44<br>LYS-44<br>HIS-161<br>ASP-162	Hydrophobic	3.66<br>3.60<br>3.83<br>3.88
			LYS-44	π-Cation	5.44

			LYS-44	H-bonding	2.32
			LYS-44	π-Alkyl	3.99
**Complex 4**	-5.12869	11112, 11110, 11107	HIS-161<br>ASN-176<br>ILE-179	Hydrophobic	3.48<br>3.99<br>3.98
			LYS-182	Alkyl	3.90
			LYS-44	C-H bonding	2.44
			ASP-162	Attractive charge	5.08
**Complex 5**	-7.20534	11095, 11093	ASP-162<br>ILE-179	Hydrophobic	3.74<br>3.89
			ASN-176	C-H bonding	2.60
			ASN-176	C-H bonding	2.46
			LYS-44	π-Cation	4.73
			HIS-161	π-Sulphur	5.00
			LEU-88	π-Alkyl	5.00
**Complex 6**	-6.48428	11112, 11107, 11110, 11110	LYS-44<br>ILE-179<br>LYS-182<br>GLN-183	Hydrophobic	3.81<br>3.82<br>3.99<br>3.85
			ASN-176	C-H bonding	2.90
**Vitamin D**	-6.7	6871, 6864, 6889,<br>6887, 6885, 6876,<br>6881, 6876	GLN-48<br>PRO-50<br>PHE-164<br>PHE-164<br>PHE-164<br>PHE-164<br>PHE-164<br>PRO-206	Hydrophobic	3.80<br>3.72<br>3.68<br>3.70<br>3.74<br>3.53<br>3.50<br>3.80
			PRO-206	H-bonding	2.39
			PHE-164	π-Alkyl	4.45
			PRO-206	π-Alkyl	4.46
**Vitamin E**	-5.7	6885, 6873, 6869,<br>6870, 6868, 6864,<br>6883, 6882, 6881	ASP-162<br>PHE-164<br>PHE-164<br>PHE-164<br>PHE-164<br>PHE-164<br>PHE-164<br>PHE-164<br>PHE-164	Hydrophobic	3.97<br>3.77<br>3.70<br>3.50<br>3.59<br>3.58<br>3.68<br>3.74<br>3.74
			PHE-164	π- π Stacking	3.69
			PHE-164	π- π Stacking	3.90
			LYS-182	π- Alkyl	4.55

(e)

**NF-kB**	**Scores**	**Ligand atoms**	**Protein residues**	**Interactions**	**Distance**

**Complex 1**	-5.37548		SER-188	Sulphur-X	3.28
**Complex 2**	-6.02001	9102	LYS-252	Hydrophobic	3.70
			LYS-221	H-bonding	2.73
			ASP-186	Attractive charges	4.00
			PRO-223	π- Alkyl	4.87
**Complex 3**	-6.00528	9124, 9127, 9100	LYS-143<br>LYS-143<br>ASP-186	Hydrophobic	3.89<br>3.97<br>3.75
			ASP-186	Attractive charges	3.16
**Complex 4**	-4.68605	9114	LEU-187	Hydrophobic	3.95
			LYS-221	C-H bonding	2.63
			LYS-221	C-H bonding	4.55
**Complex 5**	-6.16547	9098, 9119	LYS-143<br>LYS-144	Hydrophobic	3.65<br>3.75
			PRO-223	π- Alkyl	4.36
			LYS-144	π- Alkyl	5.12
			ILE-189	π- Alkyl	5.13
**Complex 6**	-5.26821	9112, 9097	PRO-223<br>LYS-283	Hydrophobic	3.43<br>3.76
			LYS-283<br>LYS-283	C-H bonding	2.62<br>3.06
			TYR-285	π-alkyl	5.39
**Vitamin D**	-6.3	5645, 5642, 5639,<br>5624, 5627	LYS-143<br>LYS-182<br>LYS-183<br>LEU-187<br>LEU-187	Hydrophobic	3.60<br>3.77<br>3.86<br>3.91<br>3.64
			HIS-140<br>ASP-219	H-bonding	2.32<br>3.14
**Vitamin E**	-5.3	5631, 5637, 5629	ARG-52<br>PRO-66<br>LYS-221	Hydrophobic	3.53<br>3.60<br>3.48
			SER-220<br>SER-226<br>ASP-251		3.25<br>2.38<br>3.20

(f)

**STAT1**	**Scores**	**Ligand atoms**	**Protein residues**	**Interactions**	**Distance**
**Complex 1**	-6.37062	21543	MET-392	Hydrophobic	3.47
			LYS-1161	Π-alkyl	4.32
			MET-392	H-bonding	3.25

			GLU-1157	Attractive charges	3.97
			MET-392	C-H bonding	2.69
			GLU-1157	C-H bonding	2.94
			GLU-1157	C-H bonding	2.60
**Complex 2**	-6.98104	21570, 21545, 21549, 21545, 21549, 21545, 21552	LYS-344<br>TYR-356<br>LYS-388<br>ASN-391<br>ALA-402<br>GLU-1157<br>LYS-1161<br>GLU-394	Hydrophobic<br><br><br><br><br><br><br>H-bonding	3.68<br>3.89<br>3.98<br>3.76<br>3.72<br>3.71<br>3.58<br>2.36
			GLU-393	Attractive charges	3.96
			LYS-1161	Alkyl	2.58
			GLU-393	C-H bonding	2.65
**Complex 3**	-6.58554	21576, 21552, 21577, 21554	MET-392<br>GLU-393<br>GLU-394<br>LYS-1161	Hydrophobic	3.94<br>3.77<br>3.49<br>3.78
			HIS-1158	Π-Sulphur	4.18
			LYS-1161	Π-Alkyl	4.20
			GLU-394	H-bonding	2.15
			MET-392	C-H bonding	2.81
			GLU-1157	C-H bonding	2.58
			GLU-394	C-H bonding	2.56
**Complex 4**	-6.18286	21556	LYS-1161	Hydrophobic	3.48
			MET-392	C-H bonding	2.89
			GLU-1157	C-H bonding	2.75
			GLU-1157	C-H bonding	2.67
			MET-1154	Alkyl	4.81
			GLU-394	Attractive charges	4.67
**Complex 5**	-6.02137	21568	LYS-1161	Hydrophobic	3.4
			HIS-1158	Π-Stacking	4.65
			GLU-394	Π-Anion	4.16
			MET-392	C-H bonding	2.74
			TYR-356	C-H bonding	2.96
			GLU-1157	C-H bonding	2.50
			MET-392	C-H bonding	2.40
			GLU-394	C-H bonding	2.62
**Complex 6**	-6.90576	21575, 21542, 21560, 21556	THR-264<br>ILE-265<br>ASN-391	Hydrophobic	3.83<br>3.51<br>3.40

			GLU-1157		3.74
			MET-392	C-H bonding	2.44
			LYS-350	C-H bonding	2.46
			GLU-1157	Attractive charges	3.51
			GLU-1157	Attractive charges	3.58
			GLU-394	Attractive charges	4.03
**Vitamin D**	-8.6	13261, 13261, 13259, 13248, 13250, 13254	LYS-150<br>VAL-153<br>GLU-268<br>GLN-271<br>GLN-272<br>TYR-1356	Hydrophobic	3.98<br>3.80<br>3.56<br>3.31<br>3.77<br>3.53
			ASN-1357<br>ASN-1357	H-bonding	2.91<br>2.35
			ARG-274	Alkyl	4.38
**Vitamin E**	-7.5	13256, 13255, 13252, 13254, 13252, 13257, 13263, 13247, 13260, 13248	GLU-268<br>GLN-271<br>GLN-272<br>GLN-272<br>GLN-275<br>LEU-351<br>GLU-353<br>PRO-442<br>GLN-1352<br>ASN-1355	Hydrophobic	3.62<br>3.71<br>3.36<br>3.82<br>3.62<br>3.78<br>3.56<br>3.84<br>3.65<br>3.76
			LEU-351	Π-Alkyl	3.99

Table S2: Best binding energies of all the complexes with targeted proteins involved in BC

Complexes	Proteins					
	AKT1	CXCR4	ER-α	IL-22R	NF-κB	STAT1
Complex 1	-5.72622	-6.28407	-5.92615	-5.63338	-5.37548	-6.37062
Complex 2	-7.04561	-6.92816	-7.72863	-6.7682	-6.02001	-6.98104
Complex 3	-6.9241	-6.71557	-2.32003	-6.55965	-6.00528	-6.58554
Complex 4	-5.74177	-7.00082	-5.27469	-5.12869	-4.68605	-6.18286
Complex 5	-6.58214	-7.43394	-7.69538	-7.20534	-6.16547	-6.02137

Complex 6	-7.74209	-8.1024	-2.3671	-6.48428	-5.26821	-6.90576
Vitamin D	-8.3	-8.0	-6.9	-6.7	-6.3	-8.6
Vitamin E	-8.6	-7.9	-8.2	-5.7	-5.3	-7.5

Table S3: shows the ADME studies output and properties of drug-likeness. All ligands included in our study follow the Lipinski rule of five with violations of low or less than 2. It was observed that complexes 1 and 4 showed the best results by satisfying Lipinski's rule of five with zero violations. These complexes have Log P values in the range of -5.46 to -5.56, blood-brain barrier permeation, and high gastrointestinal absorption and both are not substrates of permeability glycoprotein (P-gp). Their CYPs interactions indicate that both are inhibitors for CYP2C19 and CYP3A4. Both vitamins also showed favorable results indicating low gastrointestinal absorption as the only violation. The low gastrointestinal absorption observed in the analysis suggests that a greater proportion of the administered vitamins can bypass the potential degradation or metabolism within the digestive system, enhancing their availability for uptake and utilization by the body. Complexes 2 and 3 displayed two violations, while complexes 5 and 6 showed one violation.

Complexes	Pharmacokinetics									Drug likeliness					
	**GI absorption**	**BBB permeant**	**P-gp substrate**	**CYP1A2**	**CYP2C19**	**CYP2C9**	**CYP2D6**	**CYP3A4**	**Log Kp Cm/s**	**Lipinski rule of five (violations)**	**Ghose rule (violations)**	**Veber rule (violations)**	**Egan rule (violations)**	**Muegge rule (violations)**	**BA score**
Complex 1	High	Yes	No	No	Yes	No	No	Yes	-5.56	0	Yes	Yes	Yes	Yes	0.55
Complex 2	High	No	Yes	No	No	No	No	No	-3.29	2	4	1	1	1	0.17
Complex 3	High	Yes	Yes	Yes	No	No	No	Yes	-3.94	2	2	Yes	Yes	2	0.17
Complex 4	High	Yes	No	No	Yes	No	No	Yes	-5.46	0	Yes	Yes	Yes	1	0.55
Complex 5	High	No	Yes	No	No	No	No	No	-3.95	1	3	1	Yes	1	0.55
Complex 6	Low	No	Yes	No	No	No	No	Yes	-5.10	1	3	Yes	Yes	2	0.55
Vitamin D	Low	No	No	No	No	Yes	No	No	-3.00	1	2	Yes	1	2	0.55

Vitamin E	Low	No	Yes	No	No	No	No	No	-1.33	1	3	1	1	1	0.55

Table S4: Physiochemical properties and bioactivity prediction of organotin complexes

Complexes	Physiochemical properties								Bioactivity prediction					
	Log P	Polar surface are	No. of atoms	No. of Nitrogen	No. of OH and -	Violations	No. of rotations	MW	GPCR ligand	Ion channel modulator	Kinase inhibitor	Nuclear receptor ligand	Protease	Enzyme
Complex 1	1.40	6.48	21	2	0	0	5	427.23	-0.20	-0.53	-0.55	-0.73	-0.51	-0.15
Complex 2	5.71	6.48	30	2	0	2	14	553.47	-0.07	-0.37	-0.38	-0.45	-0.26	-0.09
Complex 3	6.49	6.48	36	2	0	2	8	613.44	-0.08	-0.34	-0.29	-0.39	-0.23	-0.09
Complex 4	1.98	6.48	21	2	0	0	5	447.64	-0.20	-0.53	-0.55	-0.74	-0.52	-0.16
Complex 5	4.86	6.48	27	2	0	1	11	531.81	-0.08	-0.41	-0.43	-0.50	-0.30	-0.11
Complex 6	4.24	12.9	37	4	0	1	10	675.60	-0.08	-0.36	-0.29	-0.38	-0.23	-0.09
Vitamin D	7.68	20.2	28	1	1	1	6	384.65	0.21	0.17	-0.32	1.10	0.12	0.67
Vitamin E	9.04	29.4	31	2	1	1	12	430.72	0.25	0.14	-0.21	0.41	0.28	0.24

Table S5: Toxicity prediction of complexes generated by Protox-II

Complexes	Predicted LD50 (mg/Kg)	Predicted toxicity class	Average similarity	Prediction accuracy	Hepatotoxicity	Carcinogenicity	Immunotoxicity	Mutagenicity	Cytotoxicity
Complex 1	490	Class 4	37.39%	23%	Inactive	Inactive	Inactive	Inactive	Inactive
Complex 2	264	Class 3	33.41%	23%	Inactive	Inactive	Active	Inactive	Inactive
Complex 3	490	Class 4	32.47%	23%	Inactive	Inactive	Inactive	Inactive	Inactive
Complex 4	490	Class 4	35.26%	23%	Inactive	Inactive	Inactive	Inactive	Inactive
Complex 5	264	Class 3	31.15%	23%	Inactive	Inactive	Active	Inactive	Inactive
Complex 6	750	Class 4	34.69%	23%	Inactive	Inactive	Inactive	Inactive	Inactive
Vitamin D	10	Class 2	100%	100%	Inactive	Inactive	Active	Inactive	Inactive
Vitamin E	5000	Class 5	82.25%	70.97%	Inactive	Inactive	Inactive	Inactive	Inactive

Figures:

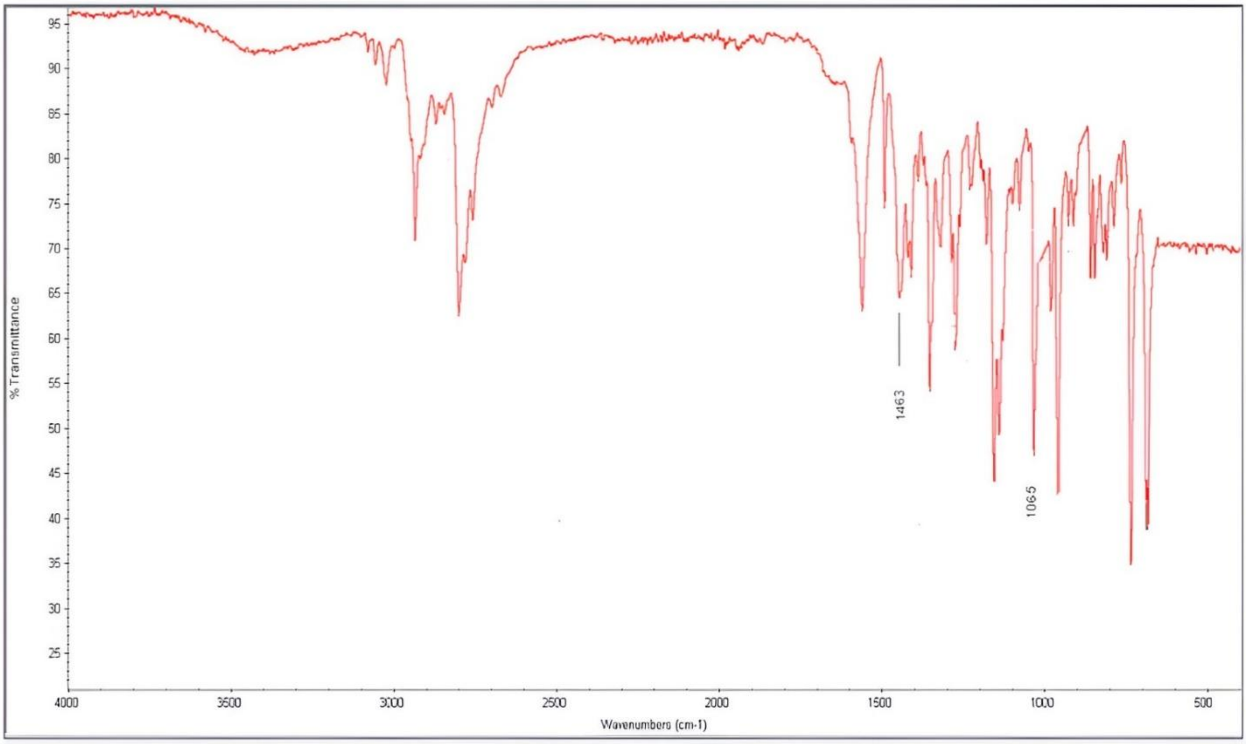

Figure S1: FT-IR spectrum of sodium salt of ligand (L-Salt)

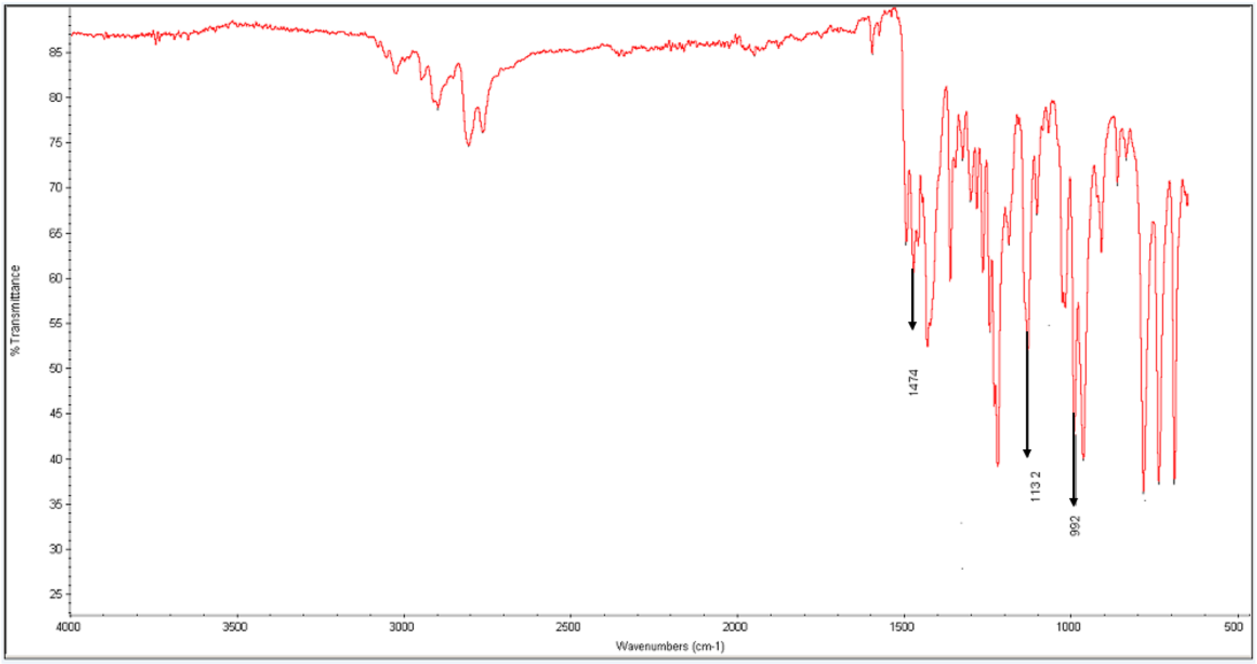

Figure S2: FT-IR spectrum of complex **1**

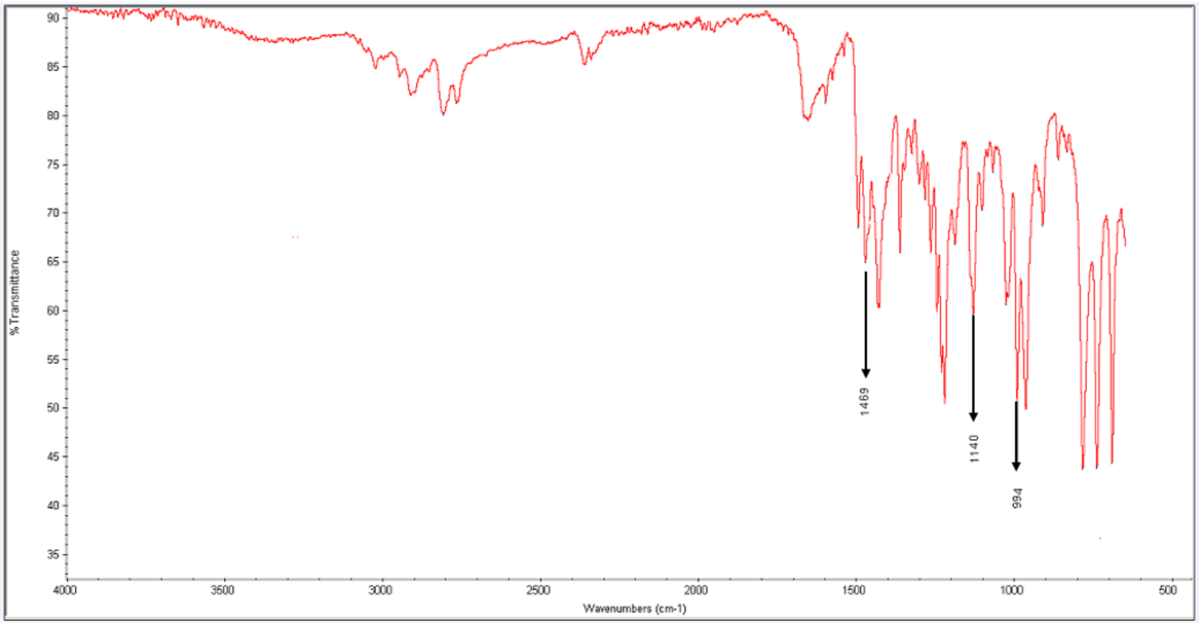

Figure S3: FT-IR spectrum of complex **2**

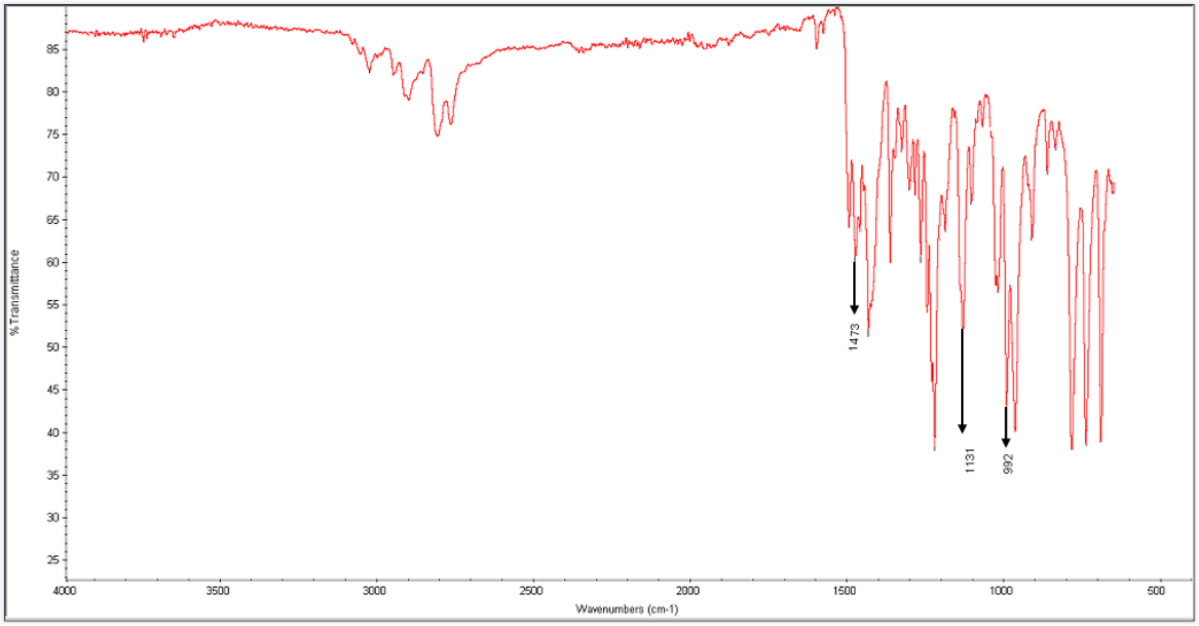

Figure S4: FT-IR spectrum of complex **3**

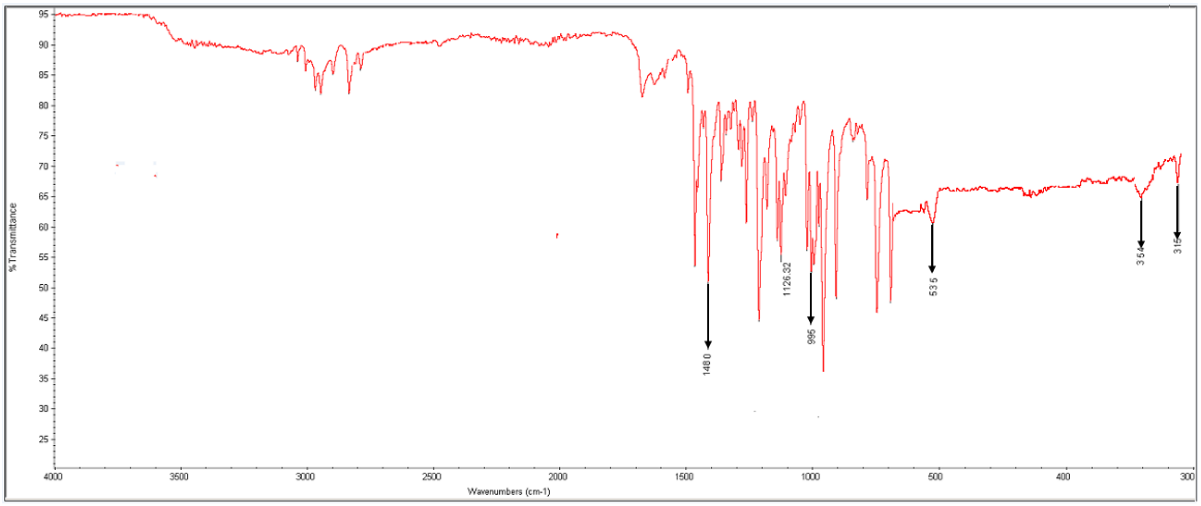

Figure S5: FT-IR spectrum of complex **4**

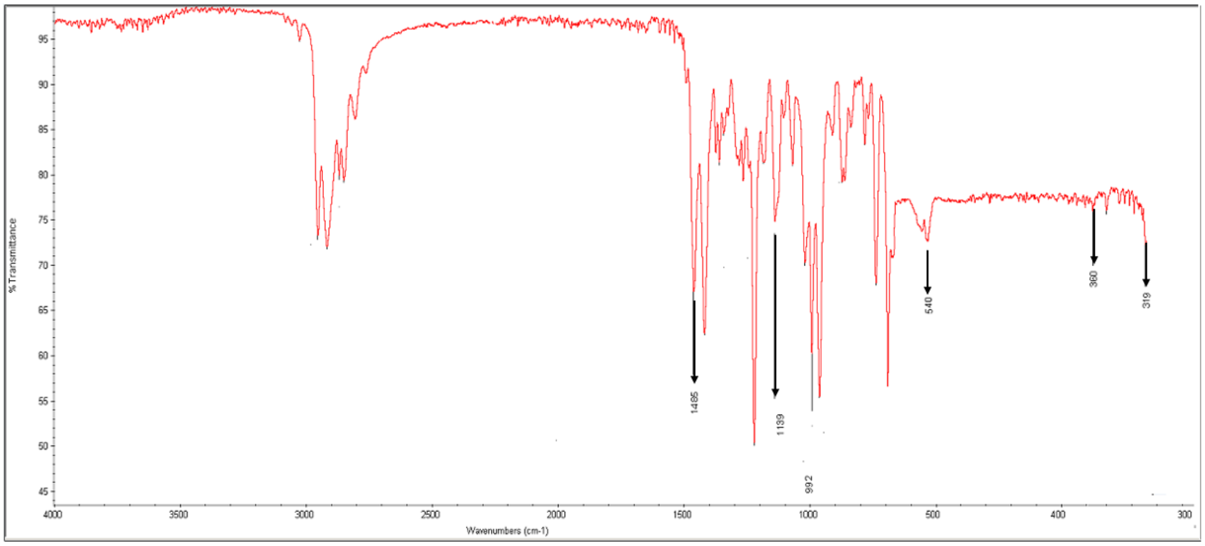

Figure S6: FT-IR spectrum of complex **5**

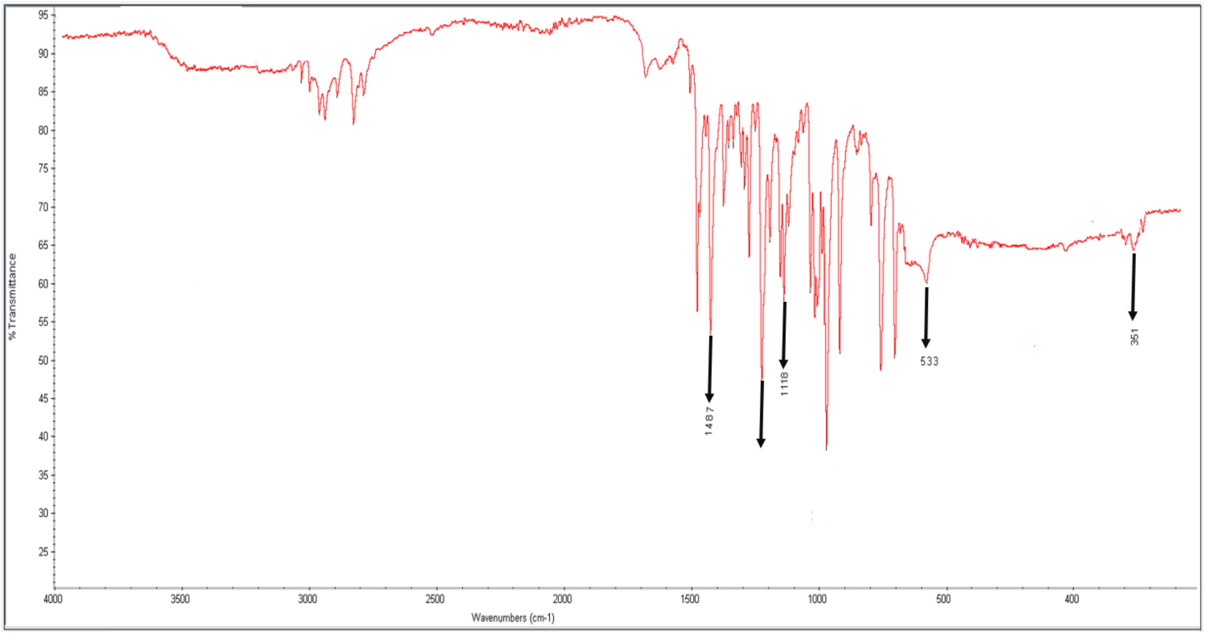

Figure S7: FT-IR spectrum of complex **6**

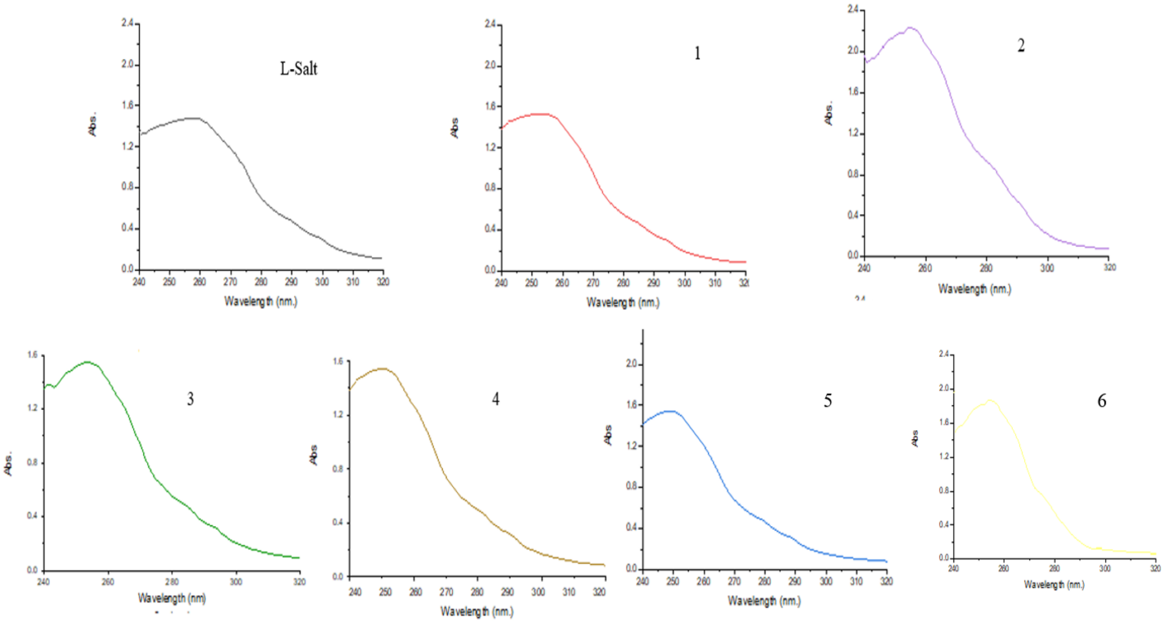

Figure S8: UV-Visible spectrum of complexes **1-6**.

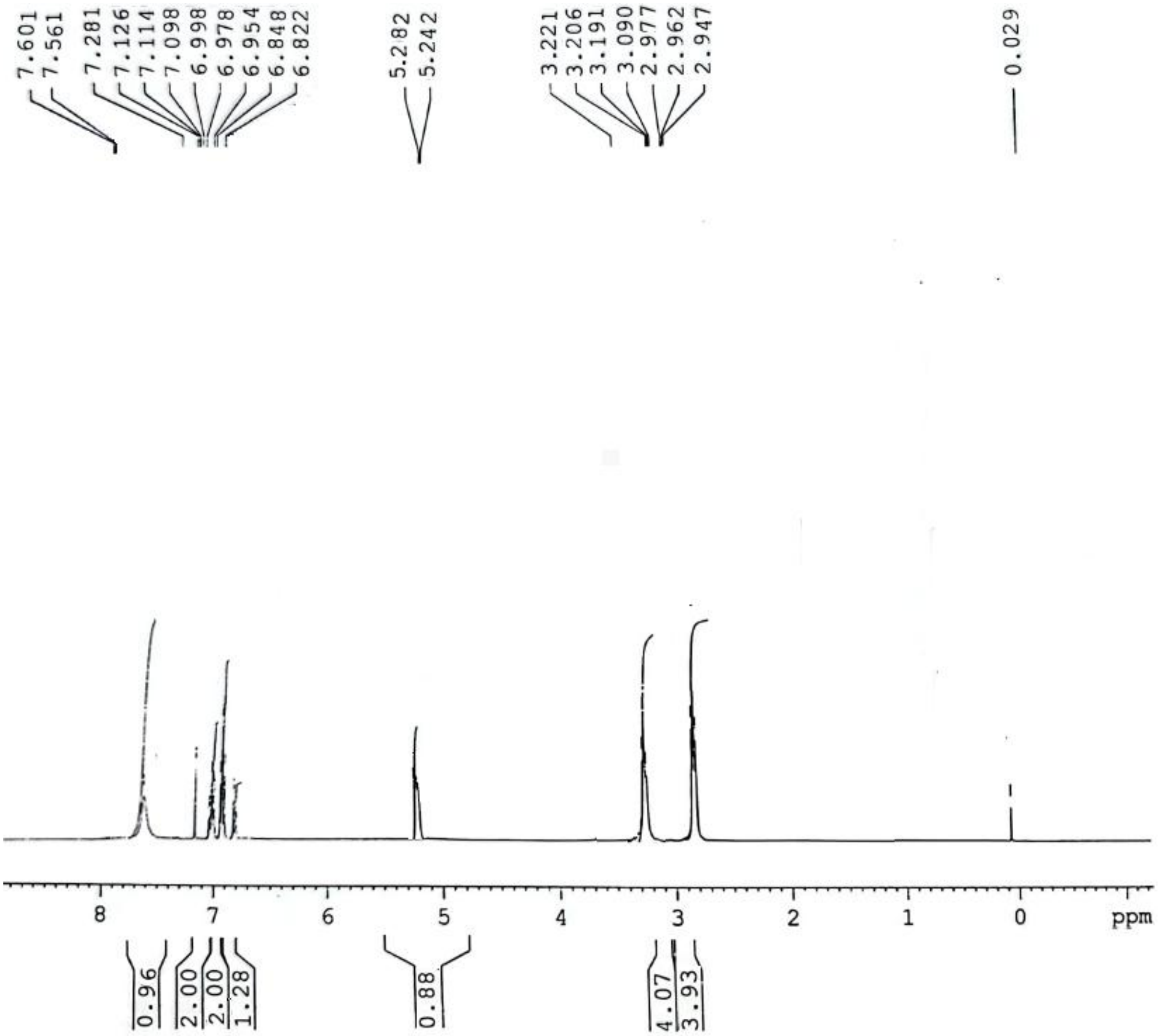

Figure S9: ^{1}H-NMR spectrum of sodium salt of ligand

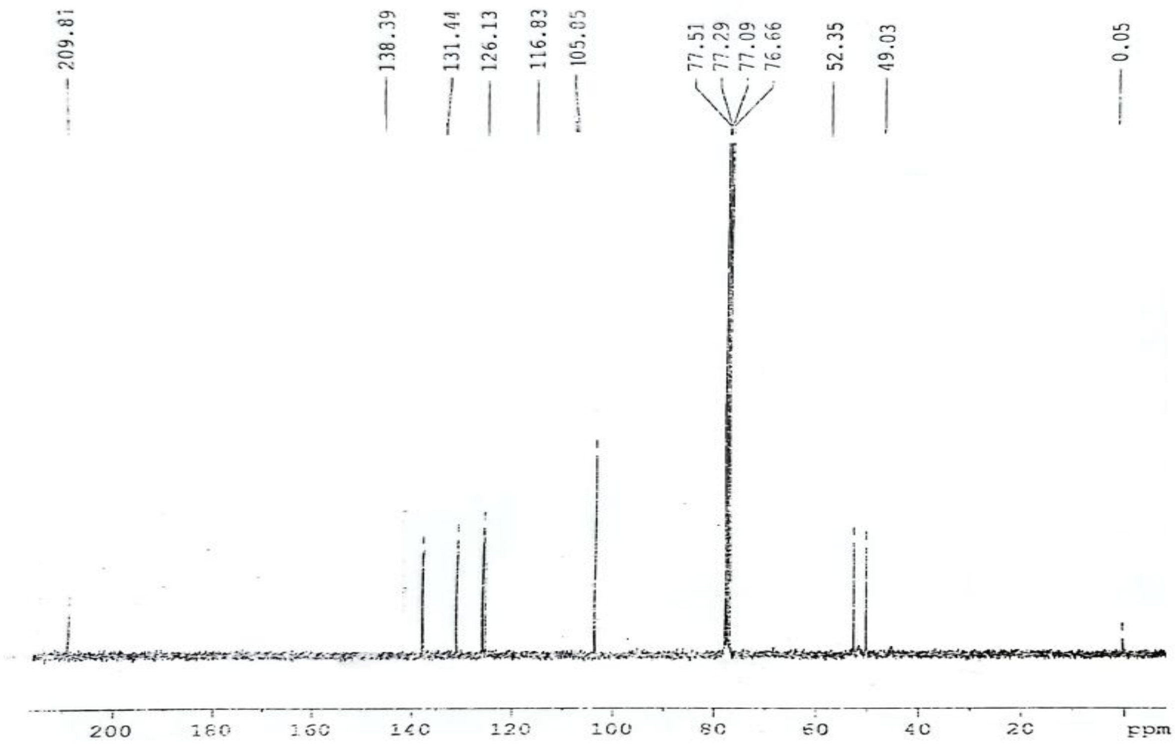

Figure S10: ^{13}C-NMR spectrum of sodium salt of ligand

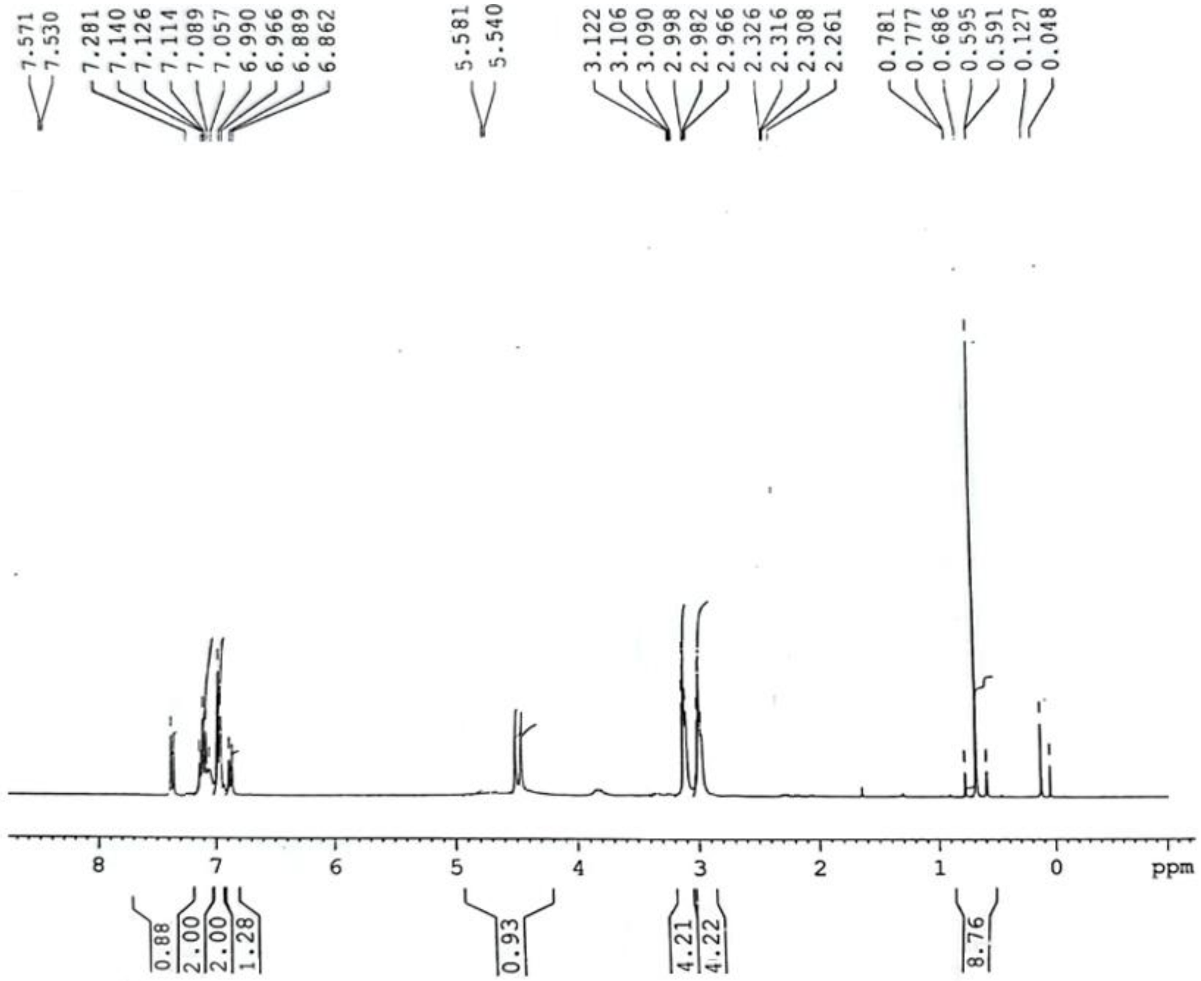

Figure S11: ^{1}H-NMR spectrum of complex **1**

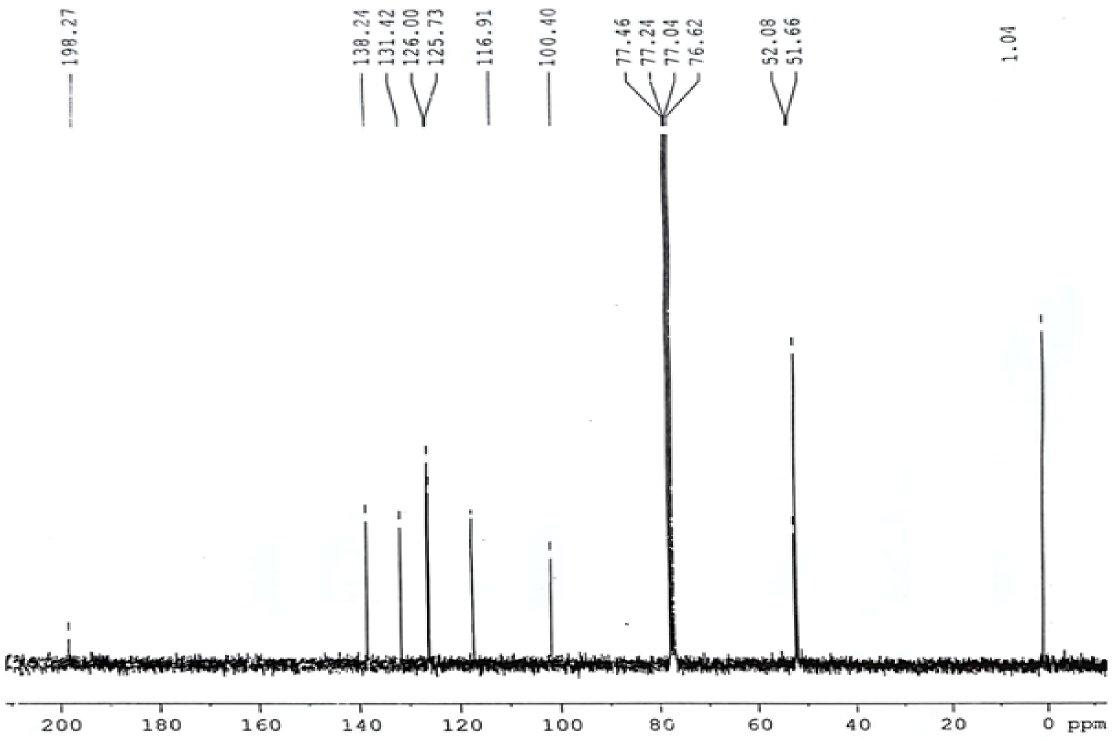

Figure S12: ^{13}C-NMR spectrum of complex **1**

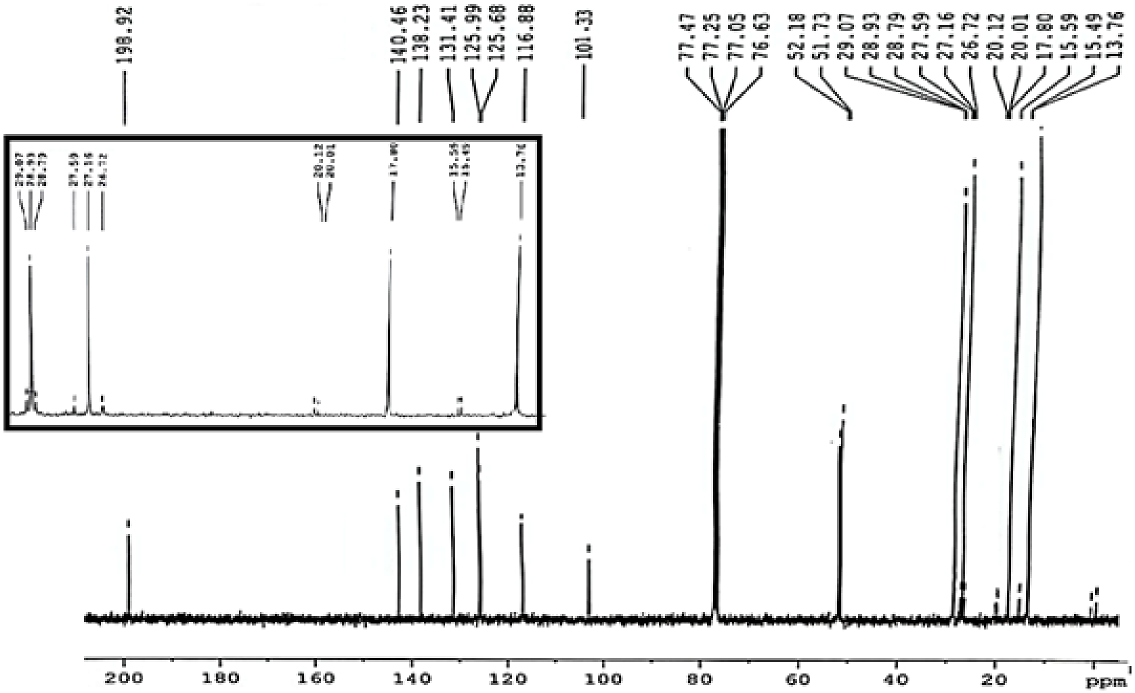

Figure S13: ^{13}C-NMR spectrum of complex 2 with an expanded view of the butyl region in the inset graph

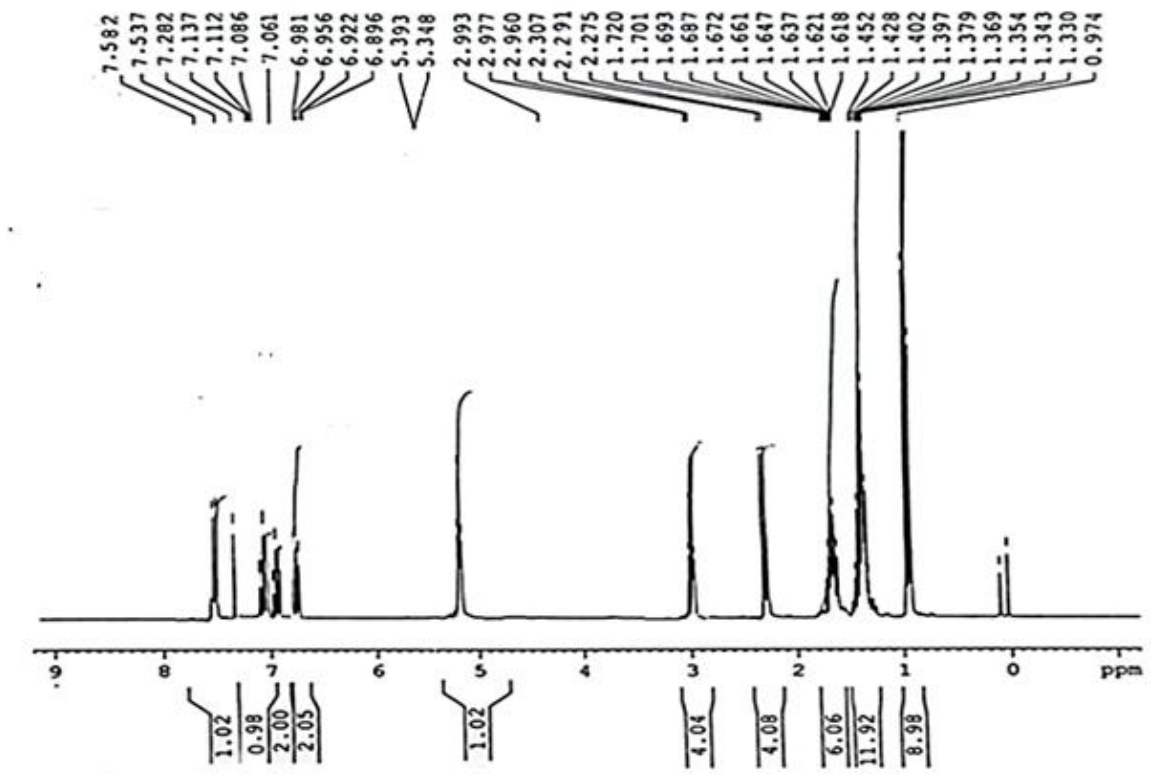

Figure S14: ^{1}H-NMR spectrum of complex 2

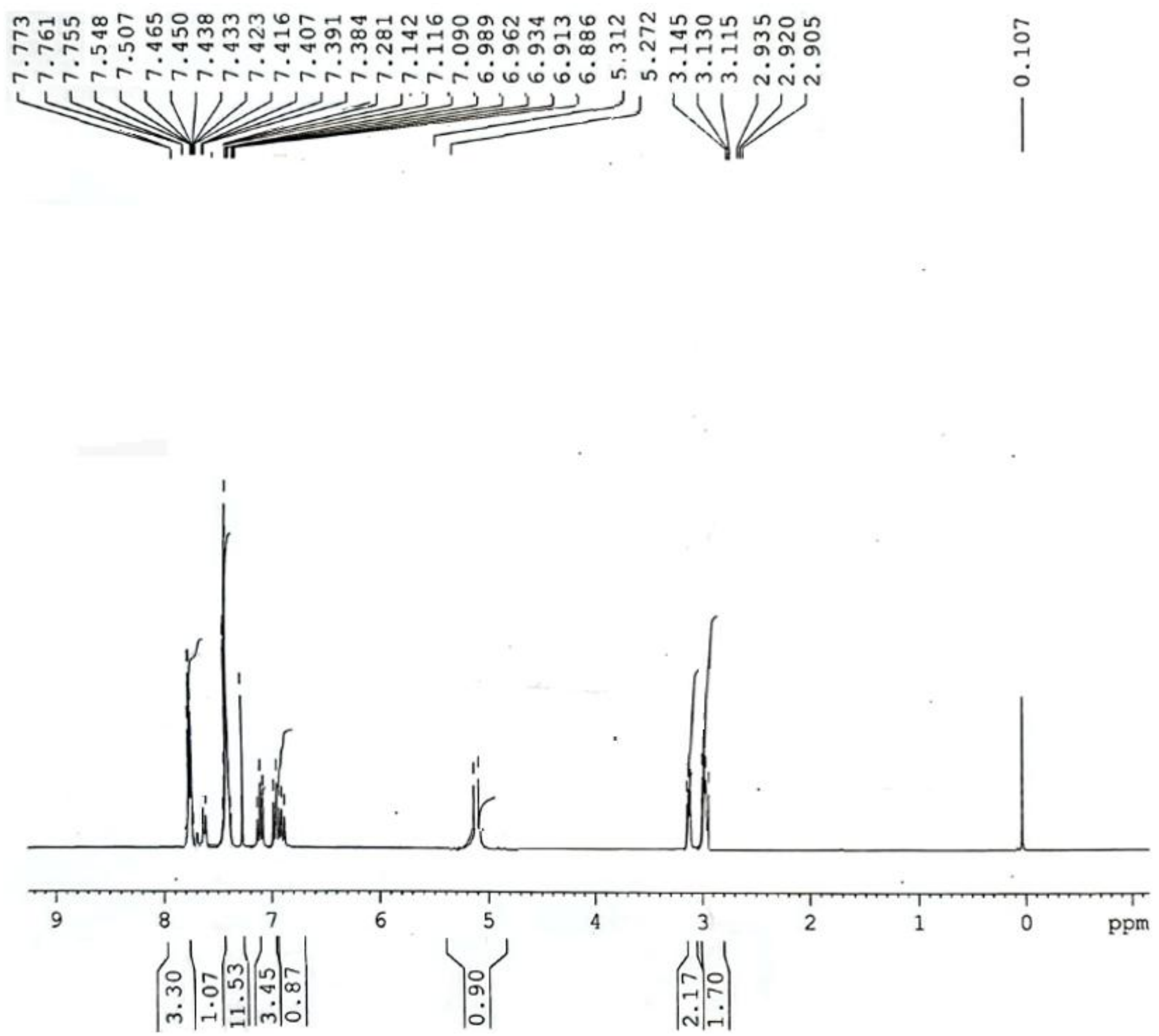

Figure S15: ^{1}H-NMR spectrum of complex **3**

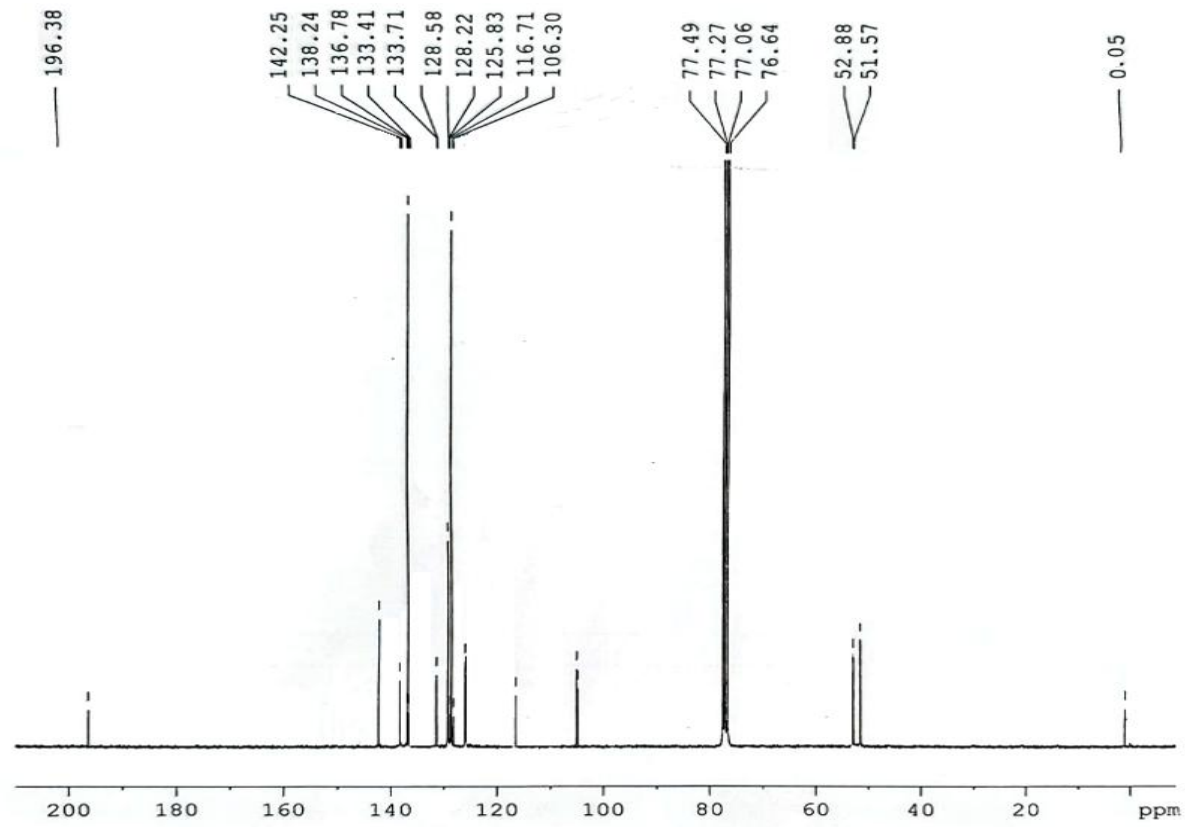

Figure S16: ^{13}C-NMR spectrum of complex **3**

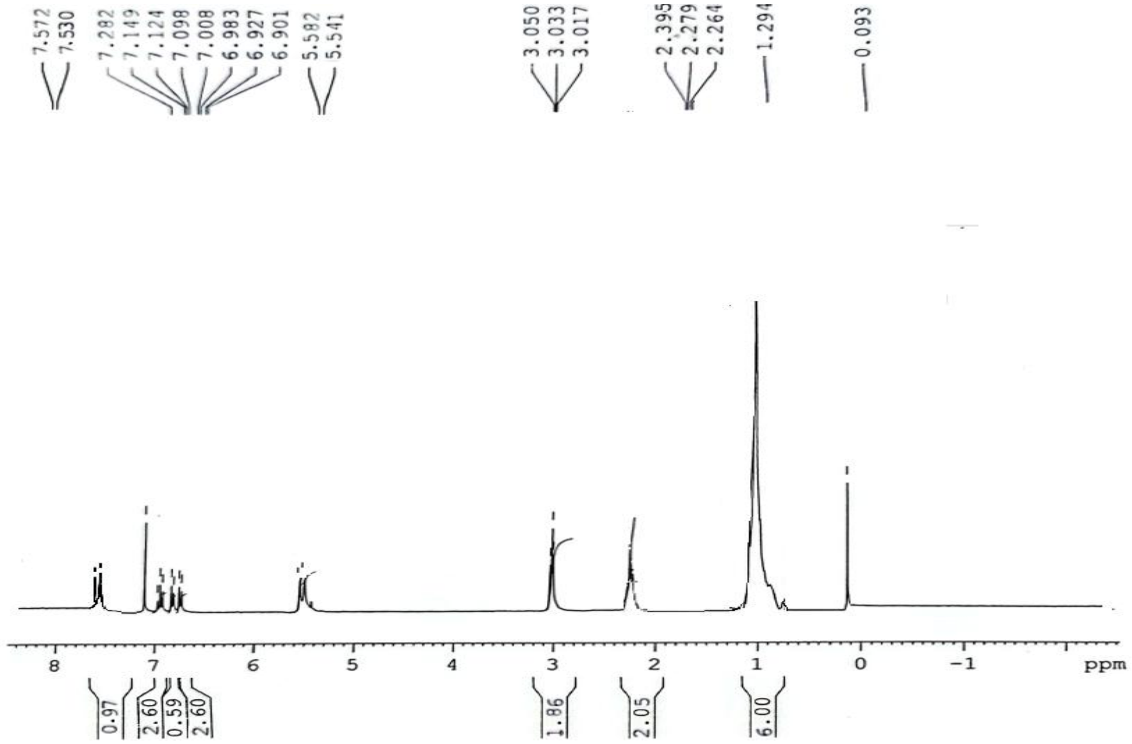

Figure S17: ^{1}H-NMR spectrum of complex **4**

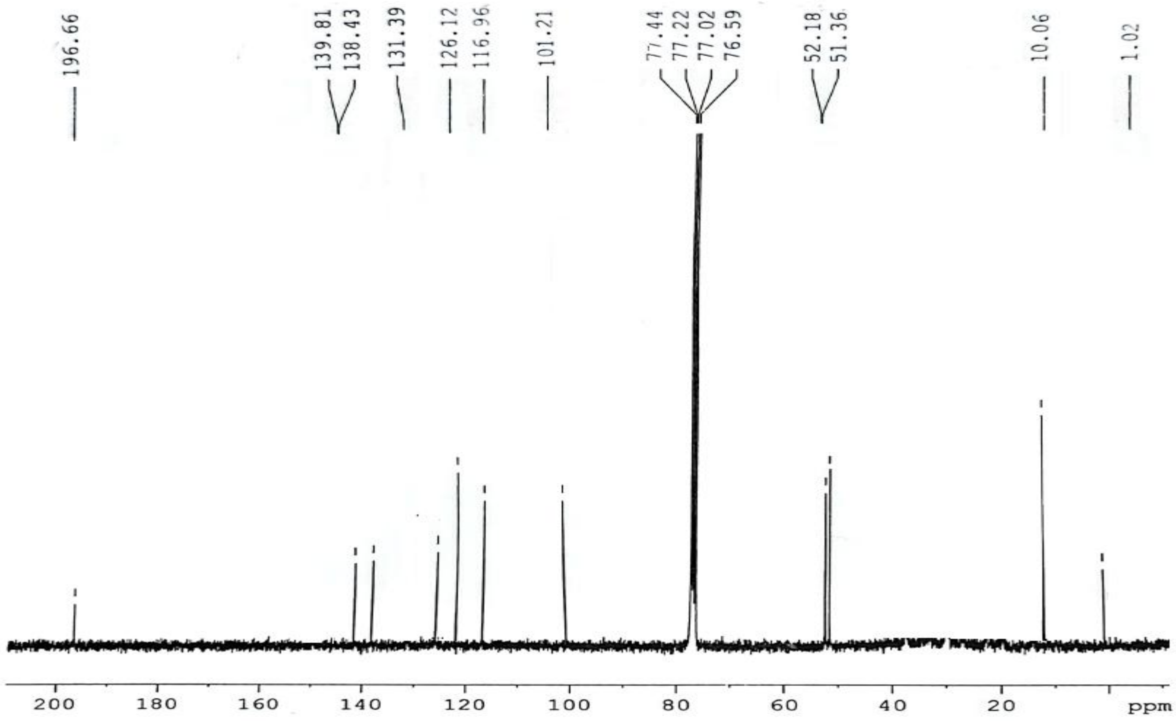

Figure S18: ^{13}C-NMR spectrum of complex **4**

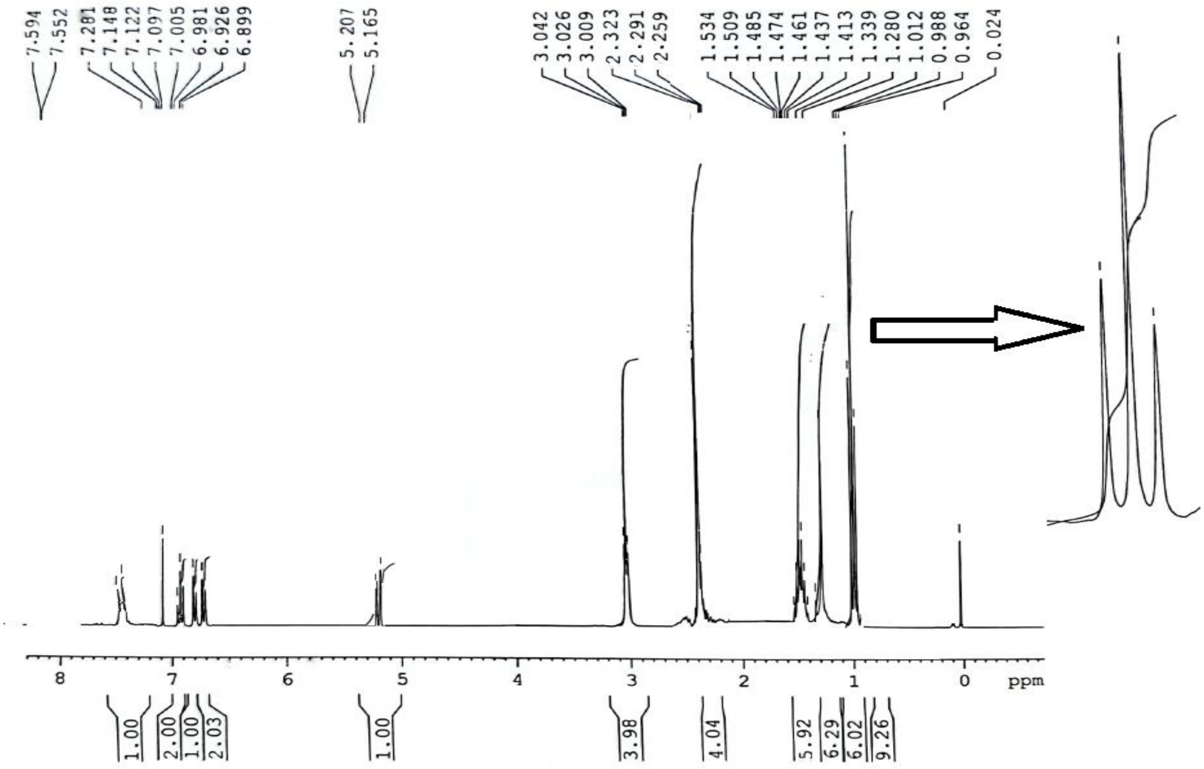

Figure S19: ^{1}H-NMR spectrum of complex **5**

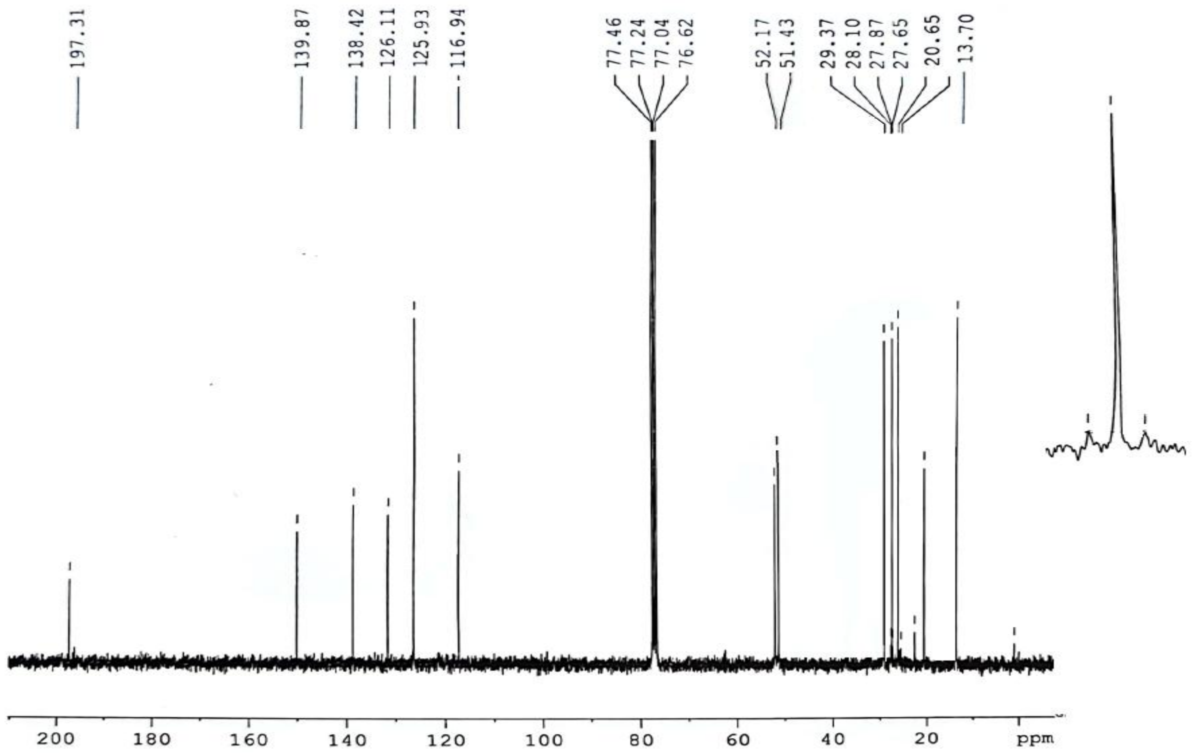

Figure S20: ^{13}C-NMR spectrum of complex **5**

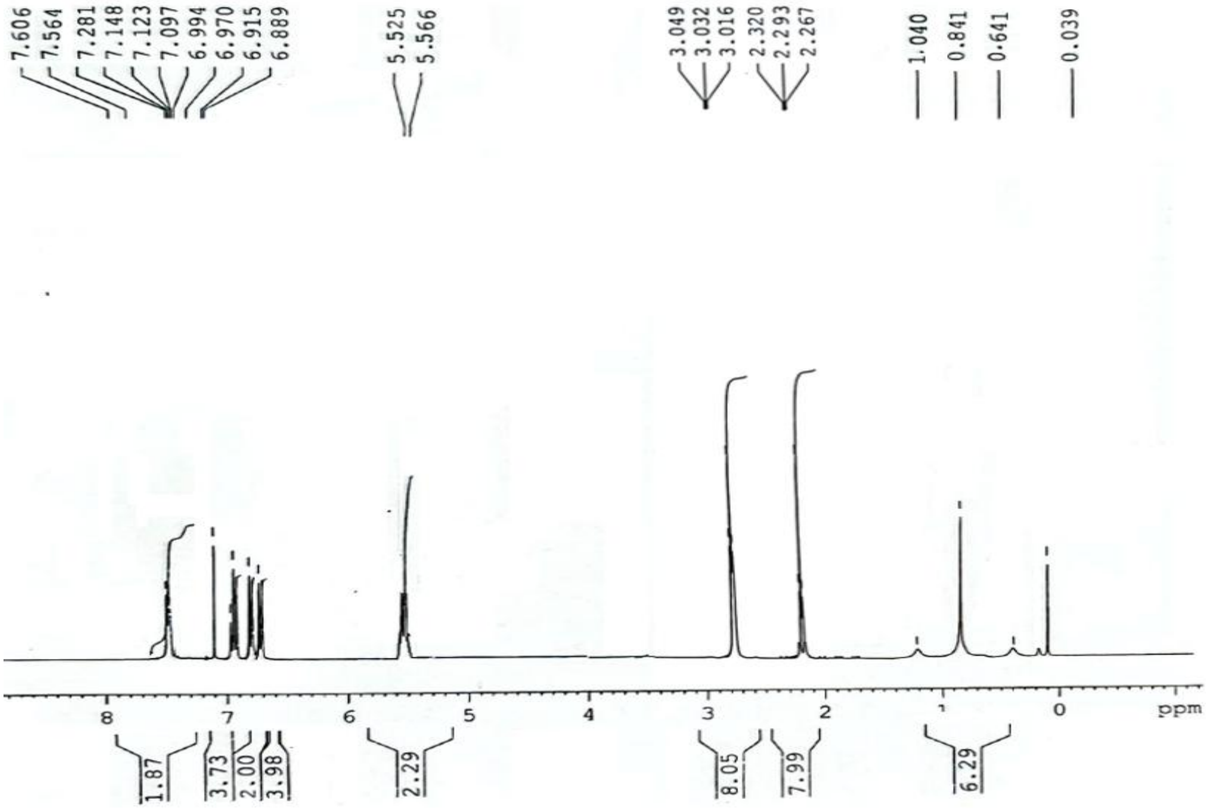

Figure S21: ^{1}H-NMR spectrum of complex **6**

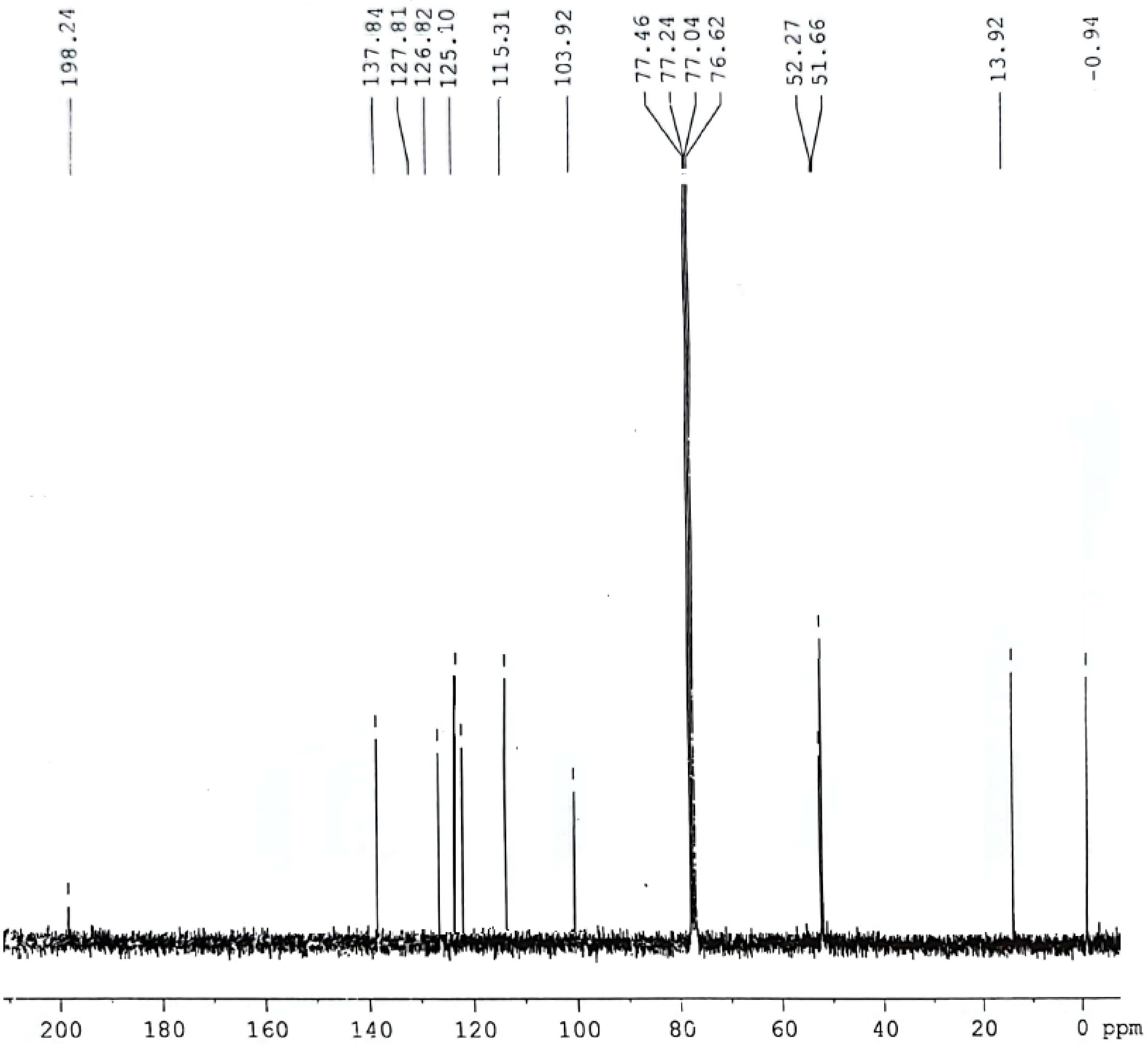

Figure S22: ^{13}C-NMR spectrum of complex **6**

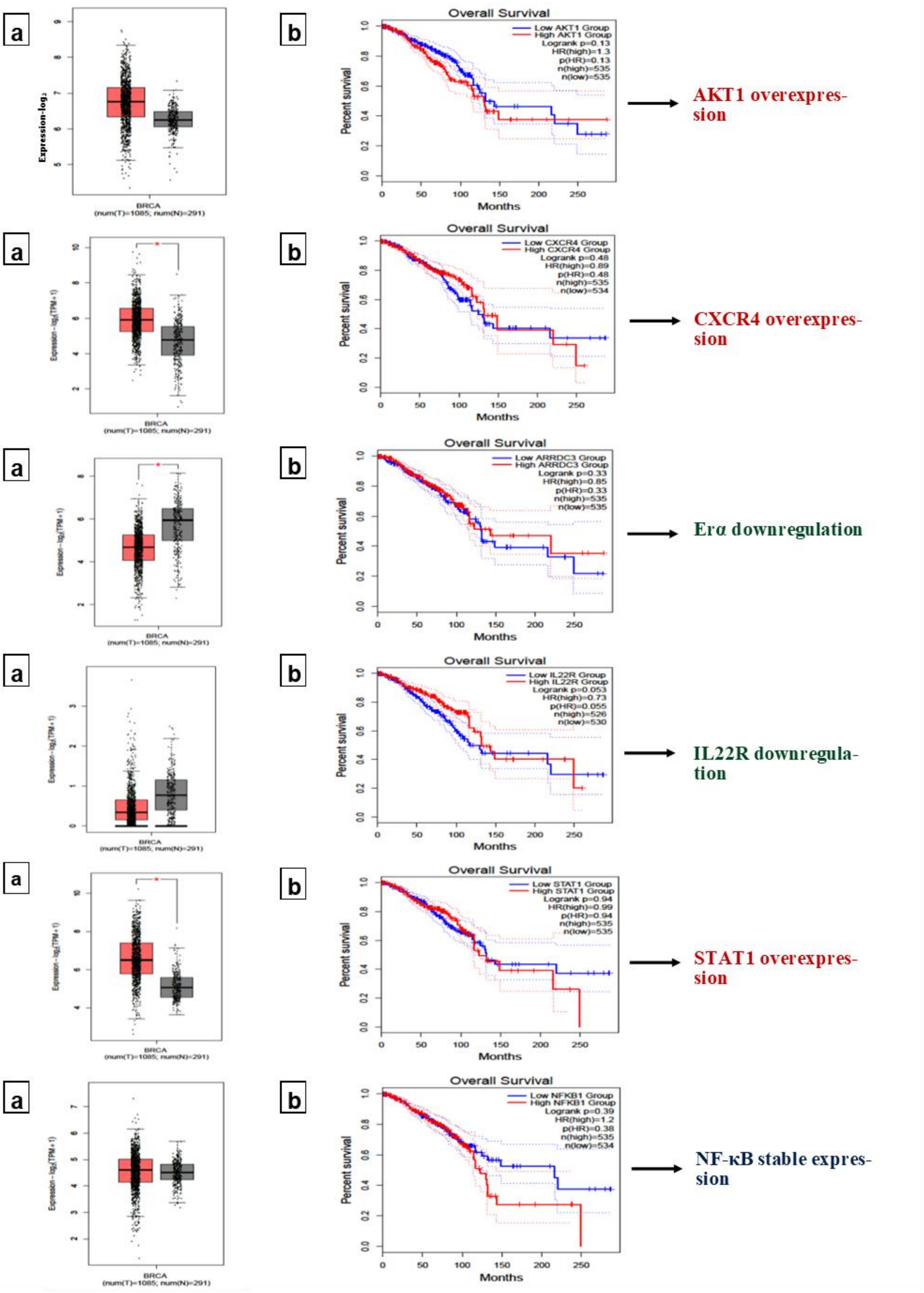
a
b
Overall Survival
Low AKT1 Group
High AKT1 Group
Logrank p=0.13
HR(high)=1.3
p(HR)=0.13
n(high)=535
n(low)=535
AKT1 overexpression
Low CXCR4 Group
High CXCR4 Group
Logrank p=0.48
HR(high)=0.89
p(HR)=0.48
n(high)=535
n(low)=534
CXCR4 overexpression
Low ARRDC3 Group
High ARRDC3 Group
Logrank p=0.33
HR(high)=0.85
p(HR)=0.33
n(high)=535
n(low)=535
Erα downregulation
Low IL22R Group
High IL22R Group
Logrank p=0.053
HR(high)=0.73
p(HR)=0.055
n(high)=526
n(low)=530
IL22R downregulation
Low STAT1 Group
High STAT1 Group
Logrank p=0.94
HR(high)=0.99
p(HR)=0.94
n(high)=535
n(low)=535
STAT1 overexpression
Low NFKB1 Group
High NFKB1 Group
Logrank p=0.39
HR(high)=1.2
p(HR)=0.38
n(high)=535
n(low)=534
NF-κB stable expression
Percent survival
Months
BRCA
(num(T)=1085; num(N)=291)

Figure S23: Expression of selected proteins in BC. (a) GEPIA database analysis demonstrated that 3 proteins, CXCR4, STAT1, and AKT1 were overexpressed, ER-α and IL-22R were underexpressed and expression of one protein (NF-κB) remained unchanged in 1085 breast cancer tissues as compared with 291 healthy tissues. (b) Prognostic significance of selected protein expression in patients with BC. Kaplan-Meier analysis was performed to estimate the association between protein expression level and overall survival time in patients included in the TCGA dataset. BRCA, breast cancer: TCGA, the cancer genome atlas.

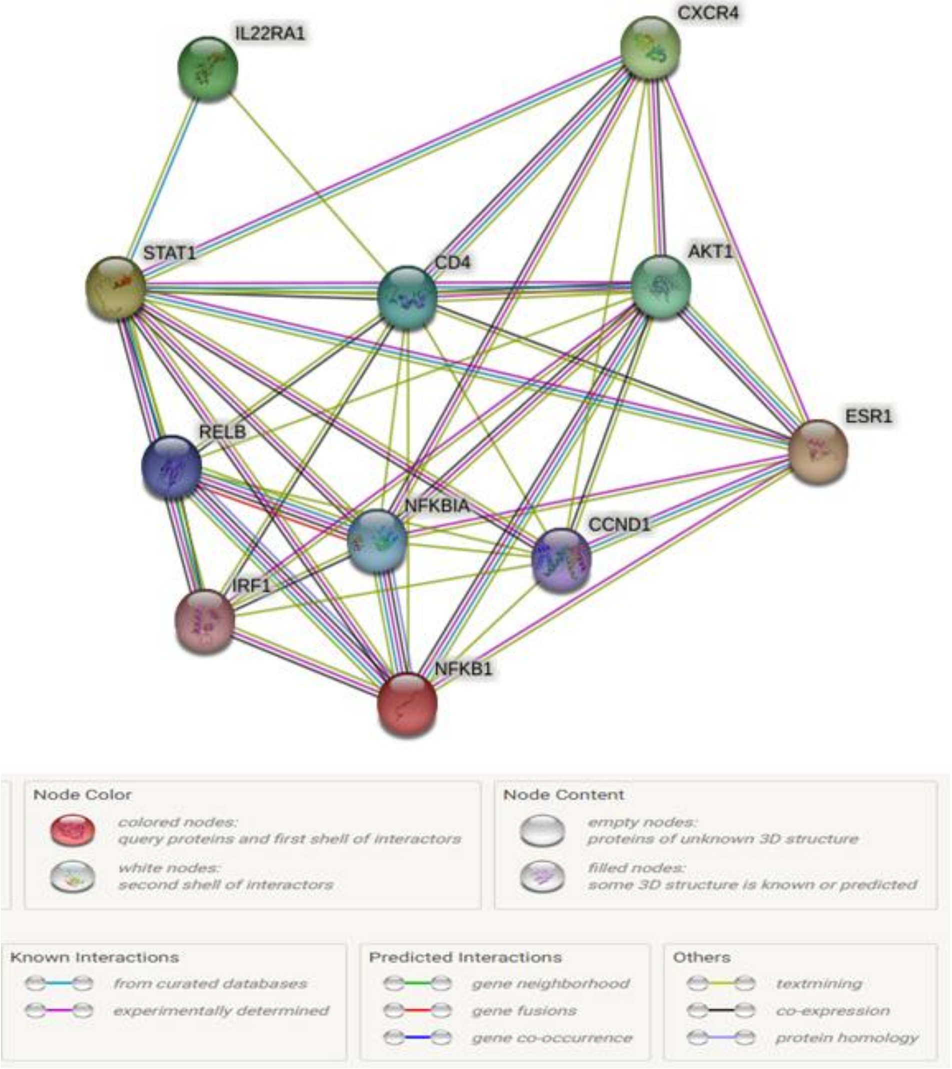

Figure S24: Protein-protein interaction map generated by STRING analysis.

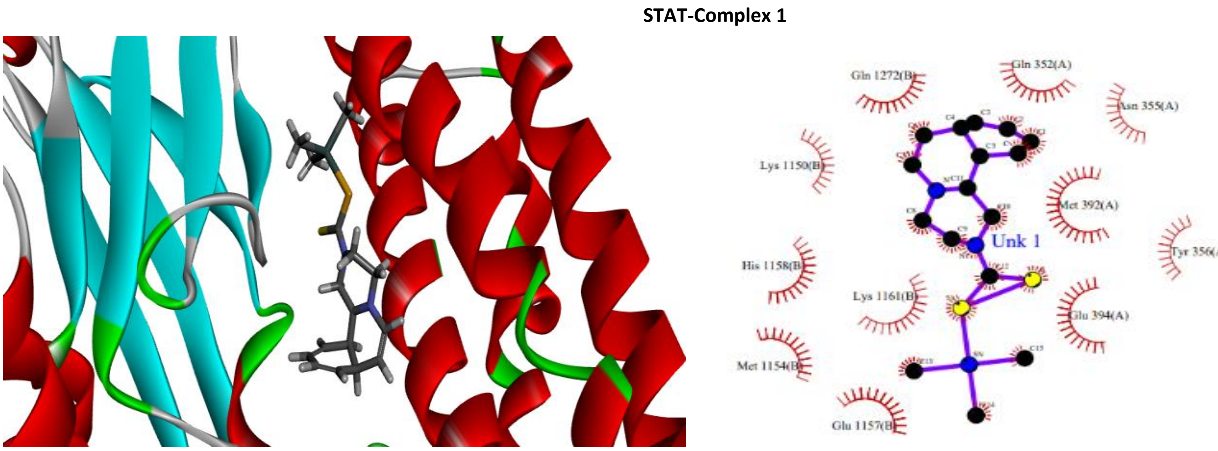

Figure S25: Docking and 2D schematic representation of Ligplot interactions for the best-docked pose of complex **1.**

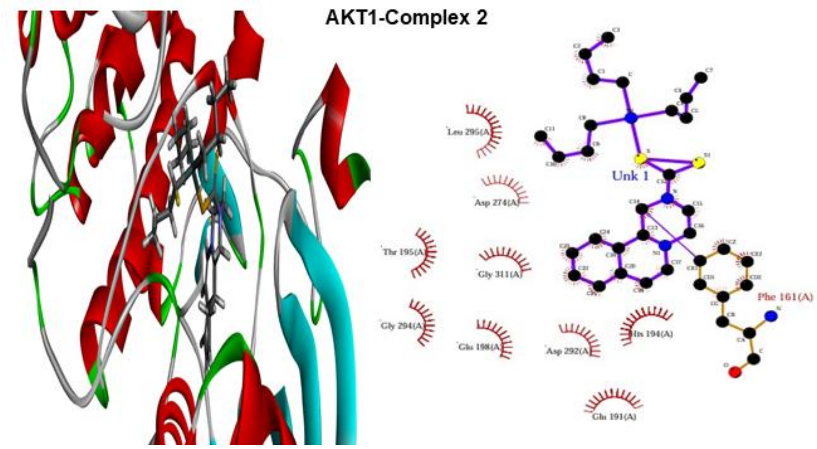

Figure S26: Docking and 2D schematic representation of Ligplot interactions for the best-docked pose of complex 2.

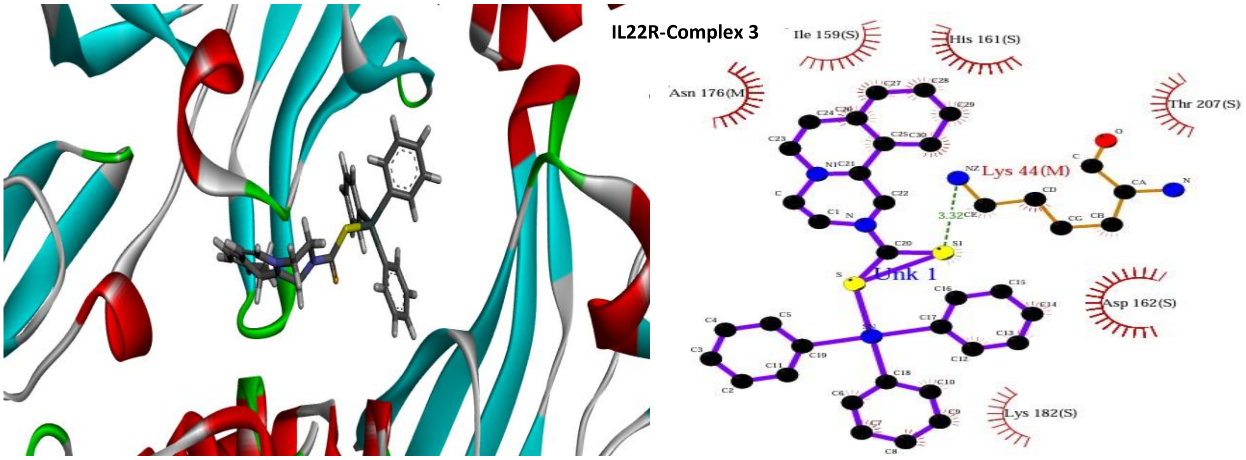

Figure S27: Docking and 2D schematic representation of Ligplot interactions for the best-docked pose of complex **3.**

CXCR4-Complex 4

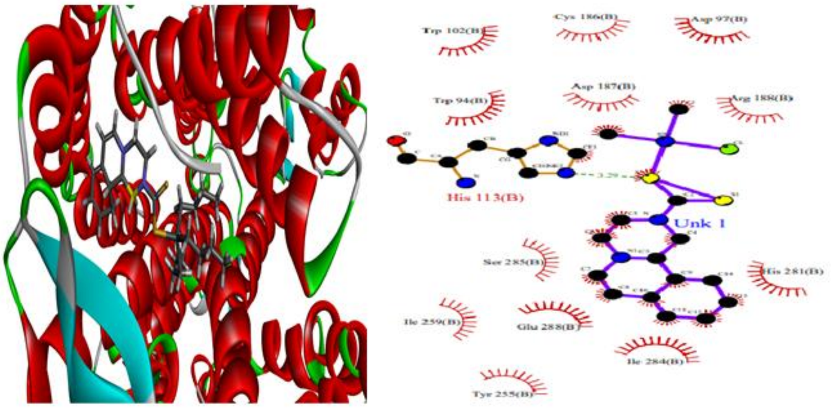

Figure S28: Docking and 2D schematic representation of Ligplot interactions for the best-docked pose of complex 4.

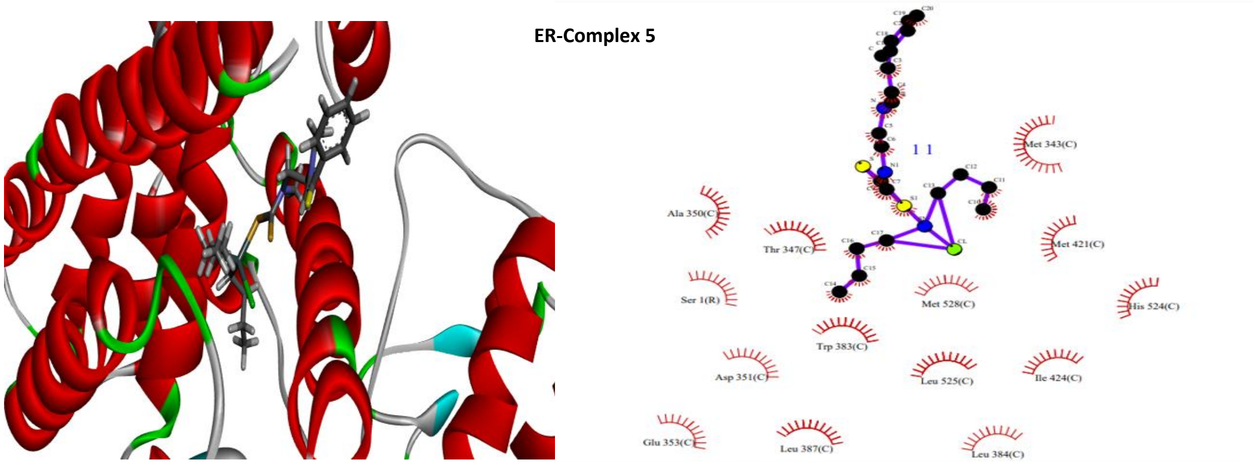

Figure S29: Docking and 2D schematic representation of Ligplot interactions for the best-docked pose of complex **5.**

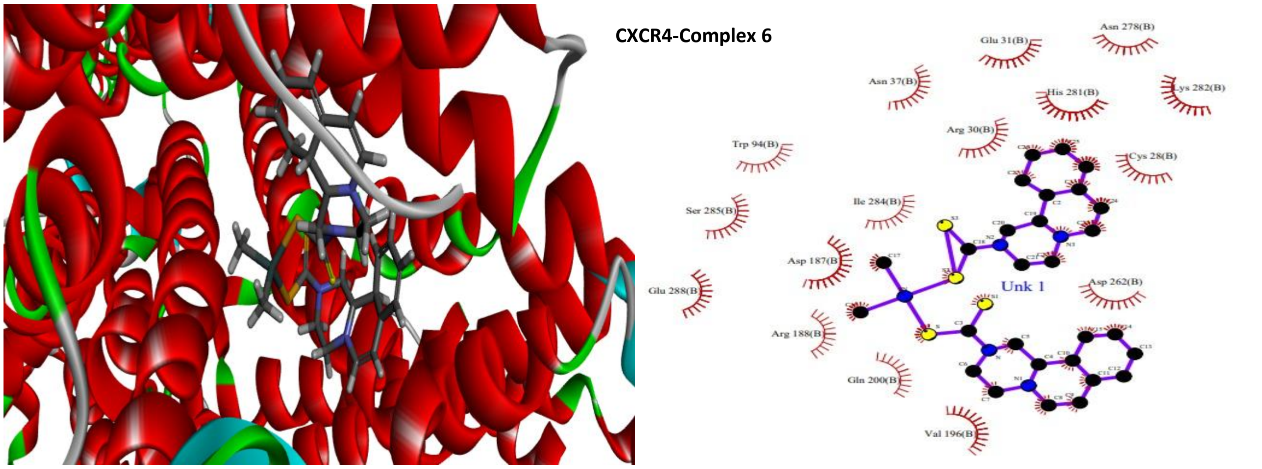

Figure S30: Docking and 2D schematic representation of Ligplot interactions for the best-docked pose of complex **6.**

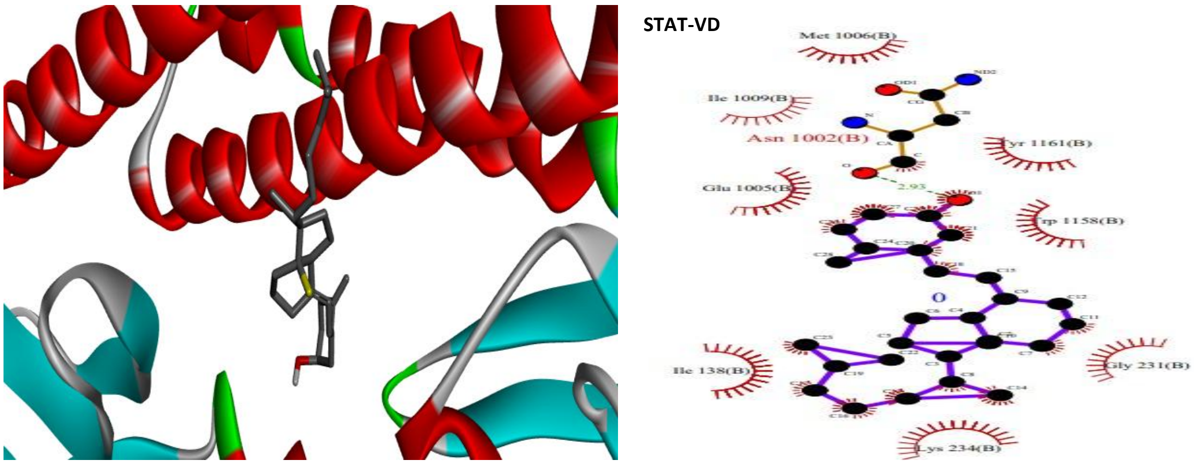

Figure S31: Docking and 2D schematic representation of Ligplot interactions for the best-docked pose of vitamin D.

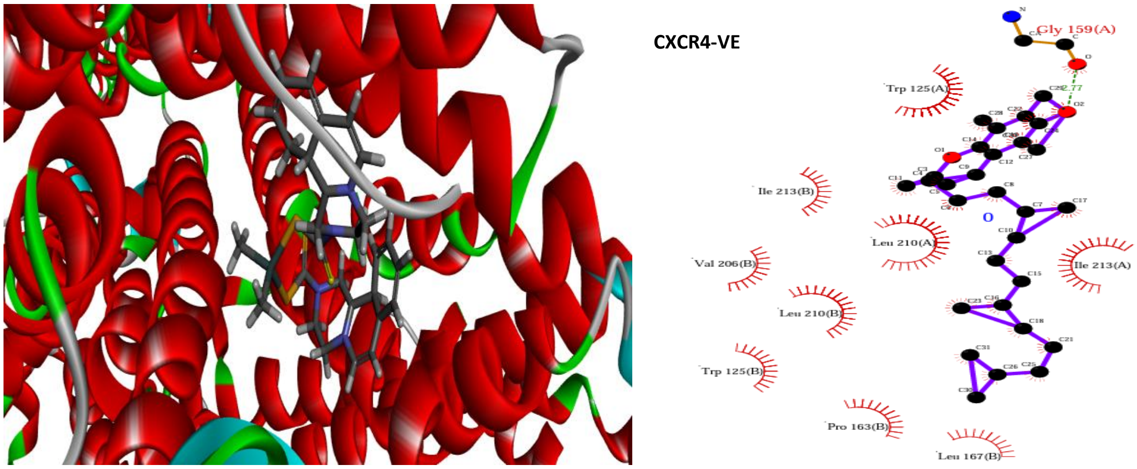

Figure S32: Docking and 2D schematic representation of Ligplot interactions for the best-docked pose of vitamin E.

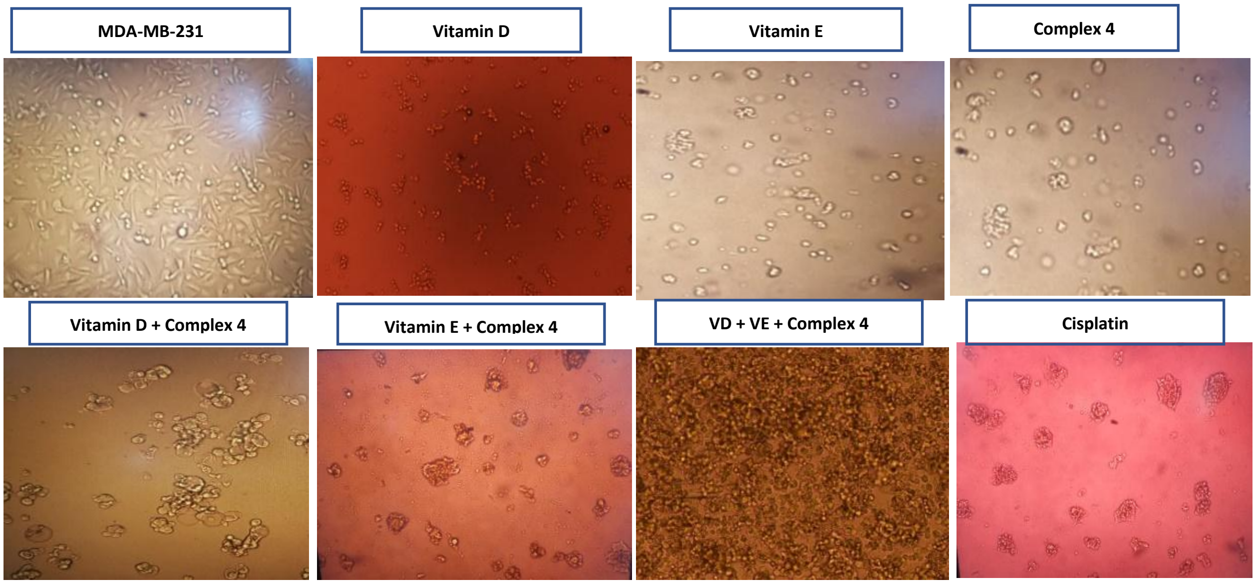

Figure S33: Cytotoxicity activity observed on MDA-MB-231 cell line by VE, VD, complex **4**, alone or in combination and cisplatin.

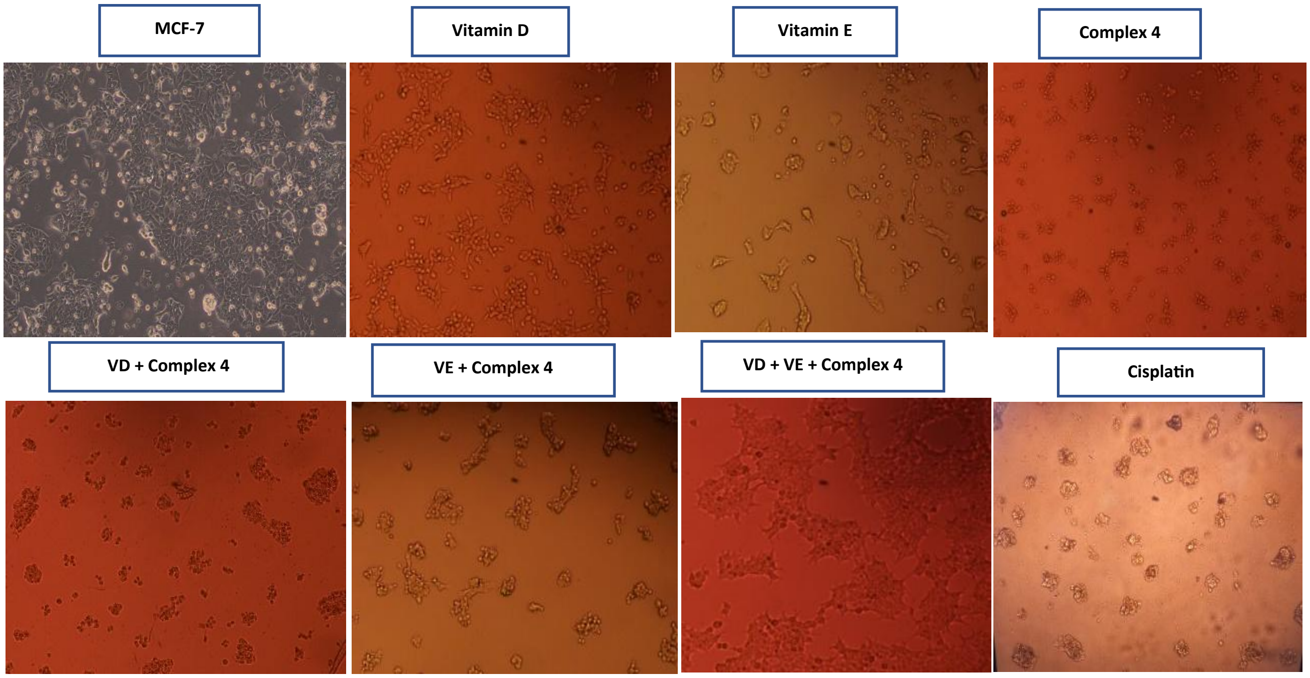

Figure S34: Cytotoxicity activity observed on MCF-7 cell line by VE, VD, complex **4**, alone or in combination and cisplatin.
